# Upper Limb Evaluation and One-Year Follow Up of Non-Ambulant Patients with Spinal Muscular Atrophy: An Observational Multicenter Trial

**DOI:** 10.1371/journal.pone.0121799

**Published:** 2015-04-10

**Authors:** Andreea Mihaela Seferian, Amélie Moraux, Aurélie Canal, Valérie Decostre, Oumar Diebate, Anne Gaëlle Le Moing, Teresa Gidaro, Nicolas Deconinck, Frauke Van Parys, Wendy Vereecke, Sylvia Wittevrongel, Mélanie Annoussamy, Michèle Mayer, Kim Maincent, Jean-Marie Cuisset, Vincent Tiffreau, Severine Denis, Virginie Jousten, Susana Quijano-Roy, Thomas Voit, Jean-Yves Hogrel, Laurent Servais

**Affiliations:** 1 Institute of Myology, Paris, France; 2 Department of Child Neurology, Centre Hospitalier Universitaire Amiens-Picardie, Amiens, France; 3 Department of Pediatrics, Division of Pediatric Neurology and Metabolism, Neuromuscular Reference Center, Universitair Ziekenhuis Gent, Gent, Belgium; 4 Reference Center for Neuromuscular Disease, Assistance Publique-Hôpitaux de Paris—Hôpital Trousseau, Paris, France; 5 Department of Pediatrics, Centre Hospitalier Régional Universitaire de Lille—Hôpital Roger Salengro, Lille, France; 6 Department of Physical Medicine and Rehabilitation, Centre Hospitalier Régional Universitaire de Lille—Hôpital Pierre Swynghedauw, Lille, France; 7 Reference Center for Neuromuscular Disease, Centre Hospitalier Régional de La Citadelle, Liège, Belgium; 8 Department of Pediatrics, Centre de références Maladies Neuromusculaires Garches-Necker-Mondor-Hendaye and Endicap U1179 INSERM—Université de Versailles-Saint-Quentin-en-Yvelines, Assistance Publique-Hôpitaux de Paris—Hôpital Raymond-Poincaré, Garches, France; 9 Thérapie des maladies du muscle strié / Institut de Myologie, Unité Mixte de Recherche S 974 Université Pierre et Marie Curie—Institut national de la santé et de la recherche médicale—Formation de Recherche en Evolution 3617 Centre national de la recherche scientifique—Association Institut de Myologie, Paris, France; Ohio State University, UNITED STATES

## Abstract

Assessment of the upper limb strength in non-ambulant neuromuscular patients remains challenging. Although potential outcome measures have been reported, longitudinal data demonstrating sensitivity to clinical evolution in spinal muscular atrophy patients are critically lacking. Our study recruited 23 non-ambulant patients, 16 patients (males/females = 6/10; median age 15.4 years with a range from 10.7 to 31.1 years) with spinal muscular atrophy type II and 7 patients (males/females = 2/5; median age 19.9 years with a range from 8.3 to 29.9 years) with type III. The Brooke functional score was on median 3 with a range from 2 to 6. The average total vital capacity was 46%, and seven patients required non-invasive ventilation at night. Patients were assessed at baseline, 6 months, and 1 year using the Motor Function Measure and innovative devices MyoGrip, MyoPinch, and MoviPlate, which assess handgrip strength, key pinch strength, and hand/finger extension-flexion function, respectively. The study demonstrated the feasibility and reliability of these measures for all patients, and sensitivity to negative changes after the age of 14 years. The younger patients showed an increase of the distal force in the follow-up period. The distal force measurements and function were correlated to different functional scales. These data represent an important step in the process of validating these devices as potential outcome measures for future clinical trials.

**Trial Registration:**

ClinicalTrials.gov NCT00993161

## Introduction

Spinal muscular atrophy (SMA) is the second most frequent autosomal recessive disorder worldwide. It is caused by homozygous absence of the *SMN1* gene [1] and results in degeneration of the spinal motor neurons. Clinical manifestations include muscle atrophy and varying degrees of weakness. The classification of the SMA is based on clinical findings, the time of onset, and maximal achieved motor function [2, 3]. The natural history of these patients has changed in the past decade due to aggressive intervention that has improved survival and quality of life [4]. In the severe form, SMA type I, children have impaired head control, never acquire a sitting position, and usually die before the age of two years. Children with SMA II achieve the ability to sit independently but never stand or walk independently. Those with SMA III achieve the ability to stand and walk independently, but about half of the patients lose this ability before the age of 18 [5, 6]. The SMA phenotype varies within each SMA type, covering a wide range of functional abilities.

Different outcome measures for motor assessment of ambulant and non-ambulant SMA patients have been proposed, but because of the broad clinical spectrum of these patients, assessments of strength have proven less than reliable. In a cross-sectional study of 70 SMA patients, Farrar *et al*. demonstrated the absence of correlation between the age of the patients and the Hammersmith Functional Motor Scale (HMFS) in type III patients. In SMA type II, a weak correlation was found, suggesting a low decline of patient’s function through time [7].

Different outcome measures for motor assessment of ambulant and non-ambulant SMA patients have been validated and are currently used in clinical trials: motor function measure (MFM) [8], gross motor function measure (GMFM) [9], HMFS [10, 11], upper limb module [12], the Egen Klassifikation (EK) scale, which employs a questionnaire regarding functional abilities and quality of life [13], and the PedsQL neuromuscular module [14]. MFM total and sub-scores show a good sensitivity to change [15]; however, a recent Rasch analysis [16] identified structural problems with nine functional motor scales used for SMA evaluation (i.e. MFM, GMFM, HMFS, Expanded HMFS, Modified HMFS-Extend, EK scale v.2, North Star Ambulatory Assessment for SMA, CHOP-INTEND and Test of Infant Motor Performance Screening Items). Issues included co-dependency of separate measures that result in duplication of information and measurement of abilities that do not accurately reflect disease progression [17, 18].

Merlini *et al*. showed that motor function is directly linked to muscle strength and that the loss of function is related to the loss of strength [19]. Objective measurement of motor strength has already proven to be reliable for SMA patients of various ages and muscle strength [20, 21], but are probably not sensitive enough for very weak patients [22]. SMA typically progresses from proximal to distal muscles with distal strength and function preserved until the last stages of the disease. Therefore, they constitute a potential target for outcome measures in clinical trials conducted in patients at an advanced stage of the disease.

The aims of our study were 1) to assess the feasibility and reliability of different strength (MyoGrip, MyoPinch) and functional (MoviPlate, MFM) measures in non-ambulant patients with SMA; 2) to assess the sensitivity to change for the same measurements over one year; and 3) to determine the sample size of non-ambulant patients with SMA needed in clinical trials to prove whether a given drug effectively stabilizes the disease.

## Patients and Methods

The protocol for this trial and supporting TREND checklist are available as supporting information; see S1 Checklist and S2 Protocol.

### Patients

The present study is part of a multicentre observational study for Upper Limb Evaluation in Non-Ambulant Patients with Neuromuscular Disorder (ClinicalTrials.gov Identifier: NCT00993161) that took place between January 2010 and January 2013. The study design and the preliminary results concerning Duchenne Muscular Dystrophy (DMD) patients were previously reported [22]. Patients with SMA participated in the study from neuromuscular centers in France (Institute of Myology, Trousseau Hospital, Paris; Raymond Poincaré Hospital, Garches; and University Hospital of Lille) and Belgium (Gent University Hospital and CHR La Citadelle in Liege). All functional and strength assessments were carried out at each recruiting center by well-trained physiotherapists. The study was approved by the French Ethics Review Board Paris VI and the Belgian Ethics Review Board of Gent and Liège. Before inclusion, all patients or their parental authorities provided signed informed consent.

Patient inclusion criteria for the multicentre observational study were: age 8 to 30 years, genetically confirmed neuromuscular disorder, and complete loss of ambulation. Exclusion criteria were major cognitive impairment, inability to stay seated for one hour, or recent upper limb surgery or trauma. In the analysis reported here, only data on patients with genetically confirmed SMA were included.

### Testing devices

The MoviPlate is a functional test designed to assess how the patient is able to generate specific repeated movements (extension/flexion of hand and fingers) also taking into consideration coordination and fatigue. MyoGrip and MyoPinch are dynamometers which measure isometric grip strength and key pinch with very high resolutions (0.01 kg and 0.001 kg, respectively)

Upper arm strength and function were assessed for the dominant and the non-dominant sides for each patient using these devices as described [22].

The Motor Function Measure (MFM) is a scale which enables an objective assessment of the motor abilities of patients with neuromuscular diseases whatever the motor deficiency. Three dimensions have been identified with factorial analysis in the validation study: D1 standing position and transfers, D2 axial and proximal motor function and D3 distal motor function [8]. Since the patients were all non-ambulant, the total score and D2 and D3 sub-scores were used for analysis.

The Brooke score, developed initially for patients with Duchenne muscular dystrophy, is a 6-level functional scale for arms and shoulders, where level 1 is best and level 6 is worse [23].

### Protocol

All patients were evaluated at baseline and at 6 and 12 months. The clinical data recorded at every visit included most recent respiratory data (e.g., forced vital capacity (FVC), type of ventilation), orthopedic and functional status (including Brooke scale), and the disease history. Time spent in the wheelchair for SMA type II patients was considered the actual age of the patients. Baseline assessment encompassed a test-retest evaluation. Both arms were tested using the MyoSet devices with a side randomly chosen for the first assessment. All patients were given between two and five trials and the maximal value was recorded on each side. For each muscle function tested, if the difference between the first two measurements was lower than 10% of the greater, the greater was accepted. If not, a subsequent measurement was made until two trials ranged within 10%. Only 5 trials were allowed for this 10% consistency criterion. The MFM was also performed at baseline and at 6 and 12 months. More details on the experimental protocol are given in a previous article [22].

### Statistical analysis

Reliability was assessed by means of intraclass correlation coefficient (ICC). ICC was computed as a single measure ICC with a two-way random-effect model (absolute agreement). Normality of the variables was assessed using a test of Shapiro-Wilk. It appeared that MoviPlate values were normally distributed, whilst MyoGrip, MyoPinch scores were not. Therefore in order to assess potential learning or fatigue effects, test and re-test results were compared using a non-parametric Wilcoxon test for paired values. Data from both dominant and non-dominant sides were pooled for each session for this analysis.

The side effect was assessed by comparing strength and functional scores on dominant and non-dominant sides at baseline using a non-parametric Wilcoxon test. Baseline values corresponded to the maximum value reached between test and retest sessions. Clinical, strength and functional baseline values were compared between SMA type II and III patients using a non-parametric Mann-Whitney test.

MyoGrip, MyoPinch, and MoviPlate baseline values on dominant and non-dominant sides were tested for correlation with clinical parameters (age, height, weight, time spent in wheelchair, FCV, Brooke score) and MFM scores at baseline using a non-parametric Spearman correlation coefficient to take into account possible non-linear relationships between variables. Correlations between strength and MoviPlate scores were also tested for each side using a Spearman correlation coefficient.

One-year follow up was assessed by comparing one-year MyoSets values with baseline values on both sides using a non-parametric Wilcoxon test. The same analysis was performed on the subgroups of patients classified by age: 14 or younger and older than 14. The age of 14 was chosen as the cutoff based on a graphical review of data evolution according to age for the MyoSet variables and MFM for all SMA types studied.

If a new clinical trial is to be designed, the key point is to know how many participants are to be added to the sample to obtain significant results for the study. The purpose of sample size calculation is to determine an appropriate number of subjects for a given study design. We assessed the sample size for a given randomized clinical trial with two independent groups using the sample size formula described previously [24]. The difference we wanted to detect was chosen as stabilization of the motor function in the treated group compared to the natural evolution of motor function in the placebo group; this was estimated based on natural history data collected in this study. The standard deviation was calculated as the standard deviation of the one year differences from the natural history group. The alpha risk was set at 5% and the power at 80%. The analysis was performed on data from patients of all ages and on the subgroup of patients over 14.

The formula used was thus:

$$\frac{2\times SD^{2}\times {\left(Z_{\alpha /2}-Z_{power}\right)}^{2}}{Diff^{2}}$$

Where with α = 5%, Z_α/2_ = 1.96 and with a power of 80%, Z_power_ = 0.842

All analyses were performed using the SPSS v.19 statistical software (SPSS Inc., Chicago, IL). The limit of statistical significance was set to 0.05.

## Results

### Clinical features

**Fig 1:**
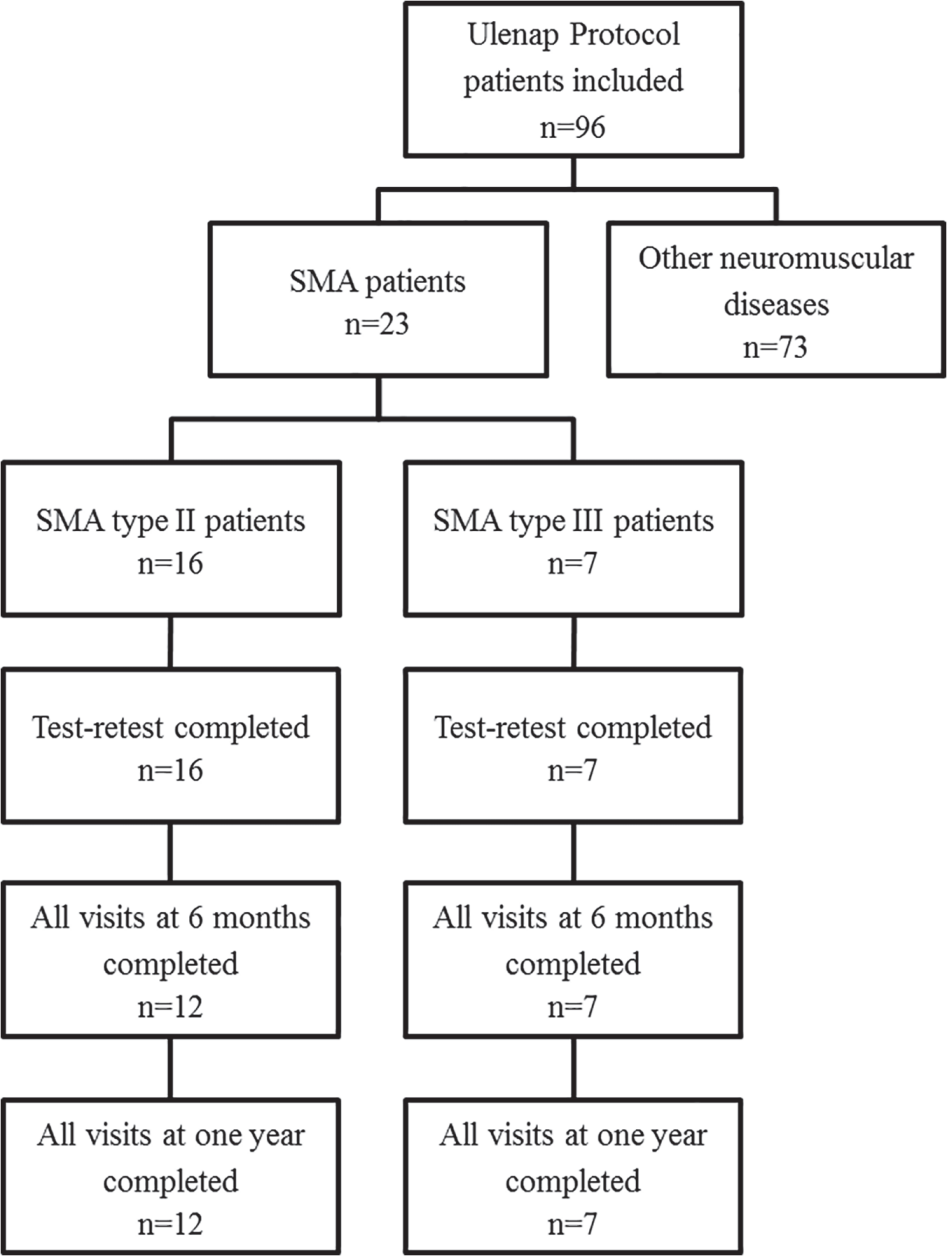
Flow chart of patients included in the clinical protocol.

A total of 23 non-ambulant SMA type II (n = 16, M/F = 6/10) and type III (n = 7, M/F = 2/5) patients were included in the study and 19 patients completed the study (Fig 1). The clinical features are presented in Table 1 and Table 2 (SMN2 copy numbers were not available for most of the patients). All patients had a homozygous deletion of exon 7 of *SMN1* gene. Four patients prematurely left the study: two refused to continue after the test-retest visit; one quit due to severe illness in the family, and another left for unknown reasons. None presented with intellectual disabilities. Seven patients were on nasal nocturnal ventilation, and one was on invasive ventilation.

**Table 1:** Clinical data of all SMA patients sorted by age.

ID	Type	Age at inclusion (years)	Weight (kg)	Height (cm)	Time spent in the wheelchair (months)	Arthrodesis	FVC (%)	NNV	Tracheo- stomy	Brooke score (#)
703	SMA III	8.3	27	121	45	no	79	no	no	2
203	SMA II	10.7	23	134	129	no	43	no	no	3
502	SMA III	10.7	57	163	NA	no	69	no	no	2
708	SMA II	11.2	49	140	135	no	NA	no	no	4
323	SMA II	12.4	25	NA	149	yes	49	yes	no	2
326	SMA II	12.5	50	159	151	yes	39	yes	no	5
321	SMA II	13.2	22	146	158	yes	22	yes	no	4
909	SMA II	13.6	37	145	163	yes	NA	no	no	2
709	SMA II	13.8	42	147	165	yes	27	yes	no	4
318	SMA II	15.3	52	161	184	yes	70	no	no	3
315	SMA II	15.4	37	141	185	yes	24	yes	no	6
320	SMA II	15.5	37	155	187	yes	16	no	no	5
512	SMA II	16.1	50	149	193	yes	24	yes	no	6
907	SMA II	16.6	47	150	199	yes	NA	yes	no	4
506	SMA III	17.5	117	154	90	NA	48	no	no	3
908	SMA II	18.9	29	130	227	yes	31	no	no	4
503	SMA III	19.9	30	175	143	yes	NA	no	no	3
107	SMA III	21.8	65	179	82	no	100	no	no	3
121	SMA II	24.8	NA	NA	298	yes	16	no	no	5
905	SMA II	26.0	40	158	312	yes	NA	no	yes	3
118	SMA III	27.4	51	162	149	no	78	no	no	3
120	SMA III	29.9	52	163	311	yes	NA	NA	NA	5
113	SMA II	31.0	35	150	372	NA	NA	NA	NA	3

FVC—Forced vital capacity (% of predicted values), NNV—nasal nocturnal ventilation, NA- not available.

**Table 2:** Clinical and functional comparison between the SMA type II and III groups.

	SMA II		SMA III	
	N	Median [Min-Max]	N	Median [Min-Max]
Age (years)	16	15.4 [10.7–31.0]	7	19.9 [8.3–29.9]
Weight * (kg)	15	37.3 [21.7–52.0]	7	52 [27–117]
Height * (cm)	14	148 [130–161]	7	163 [121–179]
Time spent in wheelchair * (months)	16	184 [129–372]	6	116 [45–311]
Forced vital capacity ** (% of predicted values)	11	27 [16–70]	5	78 [48–100]
Brooke score (#)	16	4 [2–6]	7	3 [2–5]
MFM-D2 * (%)	16	36.1 [2.6–75.0]	7	75.0 [11.1–88.9]
MFM-D3 ** (%)	16	73.8 [14.3–100.0]	7	95.2 [76.2–100.0]
MFM-Total * (%)	16	31.8 [4.2–50.0]	7	49.0 [20.8–57.3]

* p-value < 0.05 for group effect,

** p-value < 0.01 for group effect.

SMA II patients had significantly lower FVC, time spent in the wheelchair and MFM total scores and D2 and D3 subscores than the type III patients. SMA type II patients performed less well on MFM than did type III patients (-13.6). SMA II patients had a significantly lower weight and height than SMA III patients. Comparison of the two groups is presented in Table 2.

### Feasibility

The entire evaluation process including MFM, MyoGrip, MyoPinch, and MoviPlate evaluations lasted approximately 60 minutes. All patients were able to perform the MFM. One patient could not perform either the MyoGrip or the MyoPinch on the dominant side due to very severe contractures. One patient could not perform either the MyoGrip or the MoviPlate on both sides because he was too weak. One other patient could not perform the MoviPlate on either side because he was too weak, and another could not perform the MoviPlate on the non-dominant side at baseline due to difficulties in performing the task on that day. During the following visits, the patients successfully achieved the task. The lowest maximal strength detected on all visits for all patients was 0.14 kg for grip and 0.039 kg for pinch.

### Reliability

**Fig 2:**
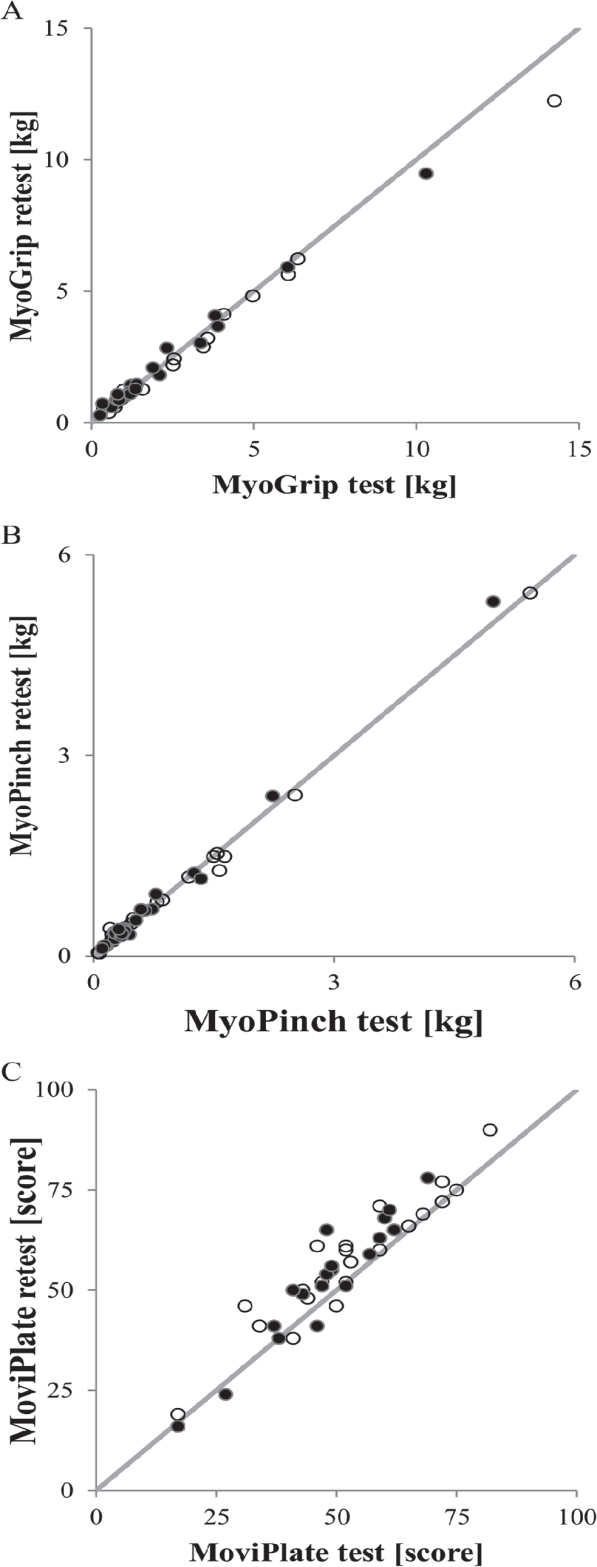
Reliability test. Reliability of test and retest sessions for (A) grip, (B) pinch, and (C) MoviPlate for all SMA patients for the non-dominant hands (dark dots) and the dominant hands (clear dots).

All measurements showed very high reliability between tests according to ICC values (all ICC > 0.95). Correlations between test and retest sessions are displayed in Fig 2 and in Table 3. There was a significant learning effect for the MoviPlate only, with a mean score increase of 4.5 between the test and retest session.

**Table 3:** Reliability parameters for SMA patients.

	N	Median value between test and retest	Median difference between test and retest [min-max]	ICC [95% CI]
MyoGrip (kg)	43	1.33	-0.01 [-2.00–0.54]	0.994 [0.990–0.997]
MyoPinch (kg)	45	0.40	0.00 [-0.29–0.31]	0.998 [0.997–0.999]
MoviPlate (#)	41	53	4 [-5-17] **	0.949 [0.730–0.982]

** p-value < 0.01.

### Dominance effect

Both groups scored significantly higher on the dominant side for grip (p = 0.020), pinch (p = 0.007), and MoviPlate (p = 0.011).

### Comparison between SMA type II and III patients

Strength performances using MyoGrip and MyoPinch and MFM scores were significantly lower in SMA type II patients for both dominant and non-dominant hands than for SMA type III patients. In contrast, there was no significant difference in the MoviPlate scores between the groups. The results are displayed in Table 4.

**Table 4:** Comparison of strength and motor function between the two groups on dominant (D) and non-dominant (ND) sides.

	SMA II		SMA III	
	N	Median [Min-Max]	N	Median [Min-Max]
MyoGrip-ND * (kg)	15	1.2 [0.3–3.9]	7	2.9 [0.9–10.3]
MyoGrip-D * (kg)	14	1.3 [0.5–6.4]	7	4.1 [0.7–14.2]
MyoPinch-ND * (kg)	16	0.4 [0.1–1.3]	7	0.7 [0.3–5.3]
MyoPinch-D ** (kg)	15	0.4 [0.1–1.5]	7	1.6 [0.4–5.4]
MoviPlate-ND (#)	13	52 [17–78]	7	63 [27–70]
MoviPlate-D (#)	14	55 [19–90]	7	69 [48–77]

* p-value < 0.05 for group effect,

** p-value < 0.01 for group effect.

### Correlations between clinical parameters and strength and motor ability

**Fig 3:**
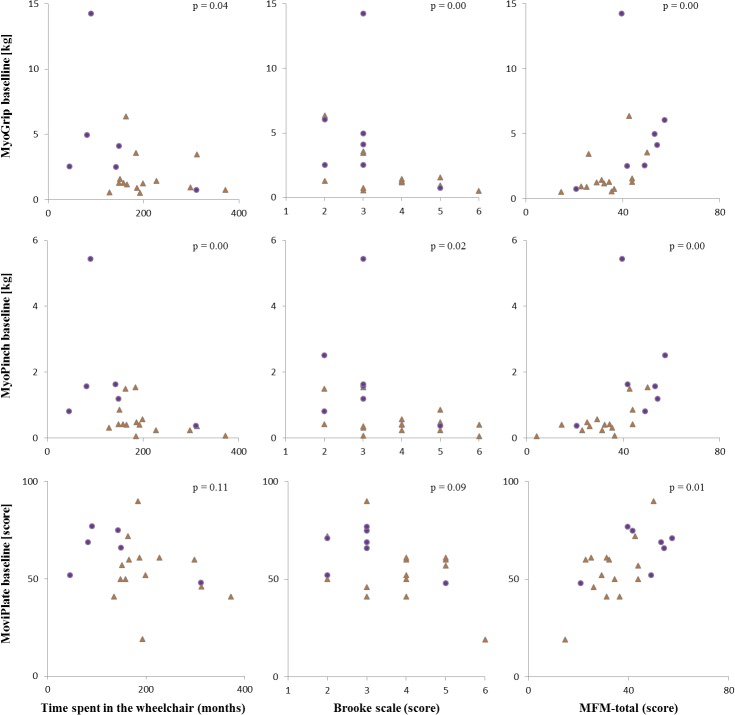
Correlations between clinical parameters and MyoSets measurements at baseline. Correlations at baseline between grip, pinch, or MoviPlate on the dominant side and time spent in the wheelchair, Brooke score, or MFM total score for SMA type II (orange triangles) and type III (purple dots) patients.

**Fig 4:**
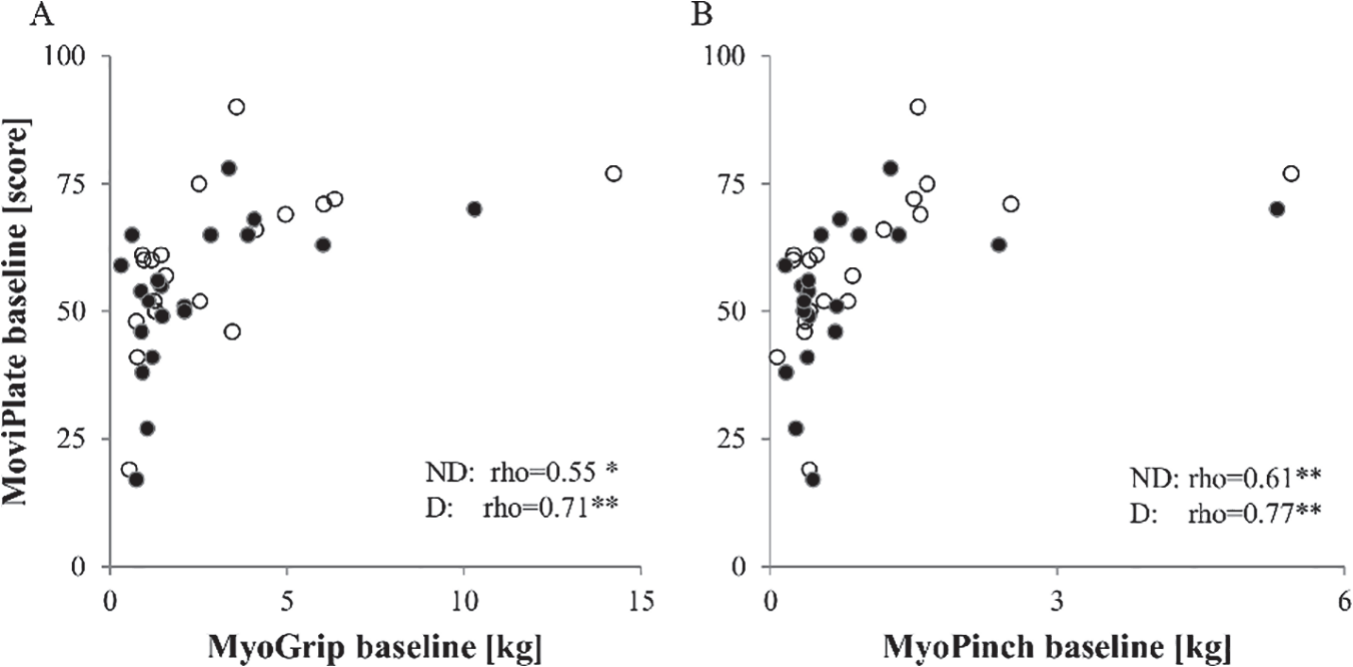
MoviPlate correlations with grip and pinch strength. Correlations at baseline between the MoviPlate scores and grip and pinch strength for all SMA patients for the non-dominant hands (dark dots) and the dominant hands (clear dots).

All tests were significantly correlated with the MFM total score. All tests with the exception of MoviPlate were significantly positively correlated with the FVC and were negatively correlated with the Brooke score. MyoPinch was significantly inversely correlated with the time spent in the wheelchair. There was no significant correlation between test scores and age, weight, or height, with the exception of MyoPinch which was correlated with weight and height (Table 5). Data on correlations are summarized graphically in Fig 3. The MoviPlate scores were correlated with both MyoGrip and MyoPinch scores (Fig 4).

**Table 5:** Correlation between MyoSets at baseline on dominant (D) and non-dominant (ND) sides, clinical and motor function parameters.

	Weight (kg)		Time spent in wheelchair (months)		Forced vital capacity (% of predicted values)		Brooke (#)		MFM-Total (%)	
	N	Rho	N	Rho	N	Rho	N	Rho	N	Rho	
MyoGrip-ND (kg)	21	0.41	21	-0.38	15	0.79 **	22	-0.69 **	21	0.72**	
MyoGrip-D (kg)	20	0.43	20	-0.47 *	15	0.74 **	21	-0.64 **			
MyoPinch-ND (kg)	22	0.44 *	22	-0.57 **	16	0.70 **	23	-0.54 **	22	0.72**	
MyoPinch-D (kg)	21	0.49 *	21	-0.66 **	16	0.66 **	22	-0.50 *			
MoviPlate-ND (#)	19	0.29	19	-0.31	13	0.30	20	-0.33	21	0.56**	
MoviPlate-D (#)	20	0.32	20	-0.37	14	0.40	21	-0.38			

* p-value < 0.05,

** p-value < 0.01.

### One-year follow-up

**Fig 5:**
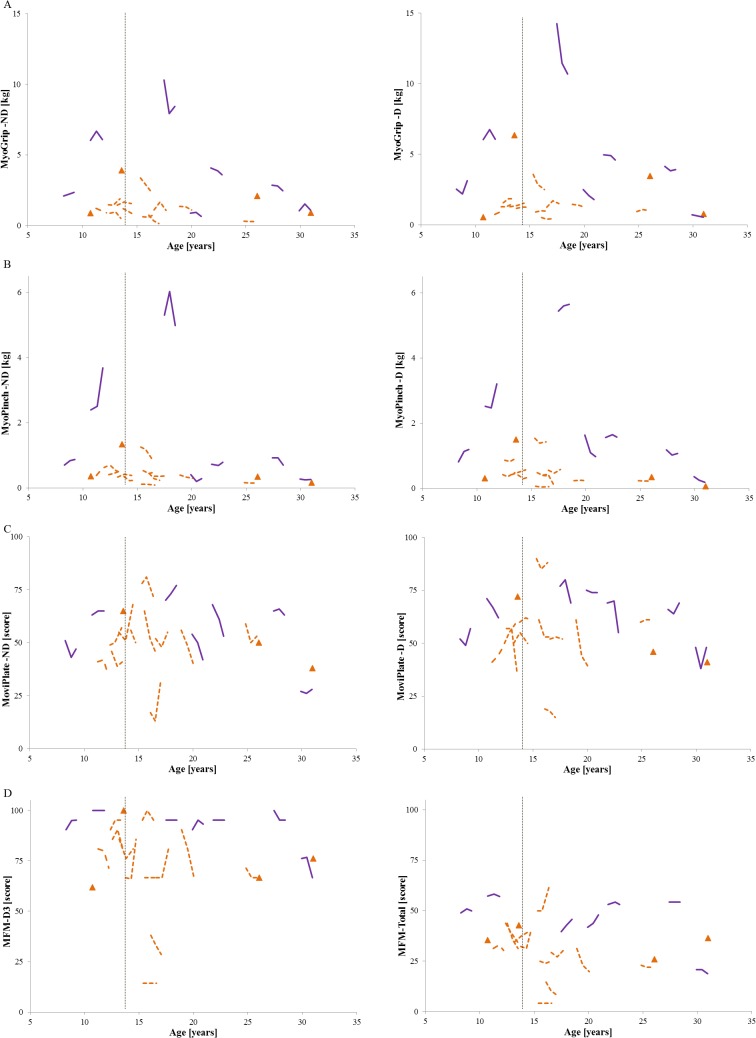
Follow-up MyoSets data and MFM scores for all SMA patients. Data at 6 months and at one year for all SMA patients for (A) grip, (B) pinch, and (C) MoviPlate on the dominant (D) and non-dominant (ND) sides and for (D) MFM-D3 and MFM-Total scores. SMA type II for whom data at 6 months and one year are lacking are marked with orange triangles; for type II patients for whom data were obtained at both time points, the orange interrupted line represents their evolution. The purple solid lines represent the evolution of SMA type 3 patients. The age of 14 is marked with a vertical dotted line.

SMA patients younger than 14 showed an increase in distal strength from baseline to one year as measured by MyoGrip and MyoPinch. In patients older than 14, significant differences between baseline and one year visit were observed for MyoGrip on both sides, for MyoPinch on the non-dominant side, and for MoviPlate score on the dominant side. No significant evolutions of the D2 subscore, D3 subscore or total score were observed for the MFM (Fig 5 and Table 6). Normalizing strength by patient’s weight as recorded during the different visits did not improve the sensitivity to change (data not shown).

**Table 6:** MyoSets and MFM evolution at one year follow-up on dominant (D) and non-dominant (ND) sides for all SMA patients, with a cutoff at 14 years.

	All patients		≤ 14 years old		> 14 years old	
	N	Median [Min-Max]	N	Median [Min-Max]	N	Median [Min-Max]
MyoGrip-ND (kg)	18	-0.2 [-1.9–0.4]*	7	0.1 [-0.4–0.4]	11	-0.2 [-1.9–0.2]*
MyoGrip-D (kg)	17	0.0 [-3.6–0.6]	6	0.2 [0.0–0.6]*	11	-0.2 [-3.6–0.3]*
MyoPinch-ND (kg)	19	0.0 [-0.4–1.3]	7	0.1 [-0.2–1.3]	12	-0.1 [-0.4–0.1]*
MyoPinch-D (kg)	18	0.0 [-0.7–0.7]	6	0.1 [-0.1–0.7]	12	-0.1 [-0.7–0.2]
MoviPlate-ND (#)	17	-4 [-19-14]	6	-1 [-6-13]	11	-6 [-19-14]
MoviPlate-D (#)	18	-1 [-22-10]	7	1 [-20-10]	11	-2 [-22-3]*
MFM-D2 (%)	19	0.0 [-25.0–16.7]	7	0.0 [-25.0–16.7]	12	0.0 [-16.7–13.9]
MFM-D3 (%)	19	0.0 [-23.8–19.1]	7	0.0 [-9.5–19.1]	12	0.0 [-23.8–14.3]
MFM-Total (%)	19	0.0 [-12.6–11.5]	7	-0.3 [-12.6–8.3]	12	0.0 [-11.5–11.5]

* p-value < 0.05.

### Sample size estimation in clinical trials

We estimated the number of patients required for a clinical trial to demonstrate a significant effect of a given intervention in stabilizing the disease during one year. This estimation was performed for all patients and for patients older than 14 years for each method. Results are summarized in Table 7. The number of necessary subjects is lower when considering patients over 14 years old.

**Table 7:** Sample size per group to include in a clinical trial to detect a stabilization of motor function on dominant (D) and non-dominant (ND) sides over a year.

	All patients			Patients > 14 years old		
	Total	SMA II	SMA III	Total	SMA II	SMA III
MyoGrip-D	57	56	55	30	36	26
MyoGrip-ND	187	14955	70	60	131	32
MyoPinch-D	15069	168	317	23	26	24
MyoPinch-ND	155404	293	1270	69	36	76
MoviPlate-D	234	501	89	79	93	75
MoviPlate-ND	104	138	65	35	34	47

## Discussion

Our study demonstrated that the use of sensitive dynamometers and a specifically designed functional test is feasible for assessing the upper limbs in almost all non-ambulant SMA type II and III patients. Measures obtained were very reliable, even in the weakest patients, who had scores far below the lower detection limit of traditional dynamometers. This is in line with our previous work in sporadic inclusion body myositis patients [25] and Duchenne muscular dystrophy [26]. The learning effect observed in the MoviPlate was similar to the results obtained in the DMD population [22]. This emphasizes the importance of planning screening sessions in clinical trials. The MyoGrip and MyoPinch show a significant correlation with clinical severity as determined by the MFM scale. We also showed that non-ambulant SMA patients tend to increase in upper limb strength before the age of 14 and to decrease afterwards. Werlauff *et al*. showed that in SMA type II patients, the decline of strength as measured by Manual Muscle Testing and function as assessed by Brooke score or EK scale decrease on a decade-time scale [27].

SMA has a very heterogeneous presentation and consists of a continuous spectrum of severities rather than well-defined types [15]. From a respiratory point of view, our population was slightly more severe than those patients evaluated in other studies. Carter *et al*. [28] reported a mean FVC in SMA type II patients of 54% at a mean age of 17±14 years; the mean FVC in our group was 32.8% in our group. For SMA type III patients, Carter *et al*. reported a mean FVC of 84% whereas that in our group was 74.8%. Nasal nocturnal ventilation was used by 46% of all our SMA type II patients compared to 38% in a 100 case series of patients aged from 3 to 50 [29]. In the previously reported series, tracheostomy was performed in 15% compared to only 6.7% in our population. The difference in the age range between our study and those in the previous studies [28, 29] may largely account for these differences.

Our study found similar values of grip strength as those reported previously for 120 ambulant and non-ambulant patients [19]. Merlini *et al*. found raw grip strength in 77 non-ambulant patients with mean age of 22.7 years at 8±11 N, which in turn must be doubled (mean: 16N). Indeed, the user manual of the Type CT 3001 Citec hand-held dynamometer recommends to multiply the value displayed by two to obtain the real strength value, which was not done in this paper (Merlini L., personal communication). This is comparable with the mean value of 24.9 N in the present study. The two series had a similar mean FVC (53% for the Merlini *et al*. cohort vs 53.8% in our group).

In our study group, the differences in age between groups of type II and type III patients were not significant. Grip and pinch strength were significantly lower in type II patients than in type III patients. The score measured by the MoviPlate was lower in SMA type II patients but this difference was not statistically significant. The relationship between strength and function is nonlinear, and changes in strength do not necessarily lead to difference in function scores, as it is believed that compensation mechanisms take place.

The strength scores were all correlated with indices of severity of the disease, such as Brooke score, with a decrease in FVC, and with time spent in the wheelchair. Interestingly, MoviPlate scores correlated with MFM score. Correlation with strength as assessed by MyoGrip and MyoPinch appeared non-linear, as previously shown in DMD patients [22, 26]. We observed a clear outlier (see Fig 3 upper middle and right column) who presented very high key pinch and handgrip strength. This patient has SMA type III and weighted 117 Kg. It is likely that this patient was basically very strong as reflected by the distal upper limb strength, which is the most preserved in spinal muscular atrophy.

SMA is described as a slowly progressive disorder. A slow decline of the pulmonary function (FVC decrement of 1.1% per year) was reported by Steffensen *et al*. [30] in 13 patients with SMA type II followed over 5 years. Vuillerot *et al*. [15] did not detect significant changes in motor function over 6 months as measured using the MFM scale but did observe a slow deterioration over a longer period (-0.9 points/year for type II and -0.6 points/year for type III). In the cohort followed by Vuillerot *et al*. substantial responsiveness in the motor function scale MFM was observed with the dimension 2 subscore (proximal and axial motricity) in patients with SMA type II [15]. These results were not reproduced in the present study (-2.5 points/year for type II and 1.6 points/year for type III) probably because of the shorter follow up duration of our study (12 vs. 21 months). Other motor function scales such as Hammersmith Functional Motor Score have failed to detect major changes in motor function over a 12-month period for SMA type II and III patients [31, 32]. Concerning strength, Febrer *et al*. did not find statistically significant changes in muscle strength using dynamometry after one year in 24 SMA patients (among them three were able to walk) [33] and changes were only detected after years of follow-up [27]. In our study, significant increases in grip strength and pinch strength in younger non-ambulant patients were observed over the course of the year, but they deteriorated in older patients. A similar motor function evolution time course has been demonstrated in other neuromuscular diseases during childhood, when the progression of the disease and the child’s growth have opposite effects on strength. For instance, in DMD patients, the distance covered during a 6-minute walk test increases before the age of 7 and decreases afterwards [34].

The age of 14 in SMA might correspond to the stage when effects of growth no longer compensate for strength loss. Data on pubertal status of patients were not collected in the present study, and it is therefore not possible to correlate our data with pubertal status (which may vary with patient) rather than with rough age. A larger group of patients must be evaluated to precisely define this cut off value and to correlate it with growth in male and female patients. To assess this point, we are currently launching a large international study including centers in France, Belgium and Germany, to assess 70 patients (clinicaltrial.gov reference will be available at the time of publication, study is currently ERB reviewed).

It is known that upper limbs strength decreases over time when assessed over long periods. Werlauff *et al*. observed a decrease in strength over a median follow-up period of 17 years (range 12–20) in 30 SMA type II and III patients with median age of 15 (range 6–53) [27]. In our study, the follow-up period was much shorter; however we also demonstrated a strength decline in patients older than 14.

Based on our data, we were able to estimate the sample size required to detect significant effects using the MyoSet devices in a placebo-controlled therapeutic trial of a compound expected to stabilize disease progression. Our data indicate that these tools should be considered for trials conducted in non-ambulant patients aged more than 14 years. To our knowledge, only one article in SMA estimated the sample size required for a clinical trial using the outcome measures reported [15]. However, not enough details on the methods are provided in this article to accurately compare samples sizes. Performing too many measures in a clinical trial may finally affect their reliability, especially in weak patients, or in patients with fatigue. Therefore, performing these tools on the dominant side only might be a good compromise to reduce testing time if strength deterioration is known to be symmetrical in the population studied. Pinch test assesses more distal muscles that are more likely to be preserved and drug respondent in the most advanced stage of the disease, but this remains to be confirmed. Given the demonstration of feasibility, high reliability, and sensitivity to negative change after the age of 14 as well as the good correlation with other clinically relevant variables, these innovative measures represent a very promising approach to assess a therapeutic intervention that aims at maintaining muscle strength and function in SMA patients. More data are required in patients below the age of 14 to better assess the relationship between strength and growth in SMA patients.

## Supporting Information

S1 Checklist TREND Checklist. (PDF)

S1 Protocol Trial Protocol (French). (PDF)

S2 Protocol Trial Protocol (English). (PDF)
