# Supplementary material for: Upper Limb Evaluation and One-Year Follow Up of Non-Ambulant Patients with Spinal Muscular Atrophy: An Observational Multicenter Trial
Source: PLoS One. 2015 Apr 10;10(4):e0121799. doi: 10.1371/journal.pone.0121799 (PMC4393256; doi:10.1371/journal.pone.0121799)
Supplement: S1 Protocol — (PDF) [file pone.0121799.s002.pdf]

PROJET DE RECHERCHE BIOMEDICALE

Développement d'un test clinique destiné à évaluer les capacités musculaires des membres supérieurs de patients non ambulatoires atteints de maladies neuromusculaires

Etude ULENAP (Upper Limb Evaluation in Non Ambulatory Patients)

Promoteur:

Association Institut de Myologie (AIM) - GH Pitié-Salpêtrière - 75651 PARIS Cedex 13

Investigateur principal:

Dr Laurent Servais, Institut de Myologie, GH Pitié-Salpêtrière

Tel: 01 42 16 99 66

Investigateurs :

Laurent Servais, Susana Quijano-Roy, Michèle Mayer, Isabelle Desguerre, Brigitte Estournet,

Scientifiques associés :

Jean-Yves Hogrel, Aurélie Canal

Plan expérimental:

Etude multicentrique sans bénéfice individuel direct

Lieux de l'étude:

- Centre de Référence des pathologies neuromusculaires – Paris Est, Institut de Myologie - GH Pitié-Salpêtrière, Paris, France.

- Service de Pédiatrie, Hôpital Universitaire Raymond Poincaré, Centre National de Référence des Maladies Neuromusculaires Garches-Necker-Mondor-Hendaye, Garches, France.

- Service de Neuropédiatrie, AP-HP, Hôpital Armand Trousseau, Paris, France.

- Service de Neuropédiatrie, APHP, Hôpital Necker-Enfants Malades, Paris, France.

- Laboratoire de physiologie et d'évaluation neuromusculaire, Institut de Myologie - GH Pitié-Salpêtrière, Paris, France.

N° d'enregistrement AFSSAPS: 2009-A00600-57

SOMMAIRE

1. INTRODUCTION *3*

2. OBJECTIFS *4*

3. METHODOLOGIE *4*

4. CRITERES D'INCLUSION ET D'EXCLUSION DES PATIENTS *5*

4.1. LES CRITERES D'INCLUSION 5
4.2. LES CRITERES D'EXCLUSION 6

5. CALENDRIER ET DESCRIPTION DES EVALUATIONS *6*

- LE TEST MOVIPLATE (ANNEXE 1) 8
- MESURES DYNAMOMETRIQUES DE FORCE MUSCULAIRE 8
- TAPING (ANNEXE 4) 9
- MESURE DE LA FONCTION MOTRICE (6, 7) (ANNEXE 5) 9

6. RECUEIL DES DONNEES *9*

7. BENEFICE ATTENDU *9*

8. DUREE D'EXCLUSION *9*

9. GESTION DES SORTIES D'ESSAI *10*

10. GESTION DES EVENEMENTS INDESIRABLES *10*

11. ENGAGEMENTS DE L'INVESTIGATEUR *10*

12. LETTRE D'INFORMATION ET CONSENTEMENT ECLAIRE *11*

13. LA GESTION DES DONNEES *12*

13.1. CONFIDENTIALITE DES DONNEES 12
13.2 ANALYSE DES DONNEES 12
13.3 ARCHIVAGE DES DOCUMENTS 12

14. AMENDEMENTS AU PROTOCOLE *12*

15. DUREE DE L'ETUDE *13*

16. RAPPORT FINAL ET PUBLICATIONS *13*

17. BIBLIOGRAPHIE *14*

18. ANNEXES *15*

ANNEXE 1 : OUTIL **MOVIPLATE** 15
ANNEXE 2: OUTILS MYOTOOLS 16
ANNEXE 3: PINCH 17
ANNEXE 4: TAPING 18
ANNEXE 5 : ECHELLE DE MESURE DE FONCTION MOTRICE (MFM) 19
ANNEXE 6 : AUTO-QUESTIONNAIRE (INDICE FONCTIONNEL DE LA MAIN) 24
ANNEXE 7 : FICHE EXPERIMENTALE 25

1. Introduction

Les maladies neuromusculaires constituent un groupe de pathologies hétérogènes quant à leur origine génétique, leur symptomatologie, leur histoire naturelle et leur pronostic. Parmi celle-ci, un certain nombre débutent dans l'enfance, voire même in utero, et se traduisent par une perte ou une absence d'acquisition de la marche. Les deux pathologies les plus fréquentes de ce sous-groupe sont la Dystrophie Musculaire de Duchenne (DMD) et l'amyotrophie spinale de type II (SMA II).

La Dystrophie Musculaire de Duchenne (DMD) est la plus répandue des myopathies de l'enfant (1). Elle touche l'ensemble des muscles de l'organisme. Elle s'exprime uniquement chez le garçon, avant 5 ans, et se traduit par la perte de la marche vers l'âge de 10-12 ans. L'utilisation des membres supérieurs se limite progressivement. Le diagnostic est porté grâce à la biopsie musculaire et est confirmé par la génétique avec la mise en évidence d'une délétion ou d'une mutation affectant le gène de la dystrophine.

L'Amyotrophie Spinale de type II (SMA II) est liée à une mutation du gène SMN engendrant une mort des motoneurones spinaux (2). Cliniquement, elle se caractérise par une perte de force proximale et distale chez des enfants, filles et garçons ayant acquis la station assise mais pas la marche.

D'autres pathologies moins fréquentes, telles les sarcoglycanopathies, les alpha dystroglycanopathies, certaines myasthénies ou myopathies congénitales, les dystrophies mérosine négative, la dystrophie d'Ullrich, voire certaines formes de laminopathies peuvent également s'exprimer dans l'enfance et se traduire par une perte ou une absence d'acquisition de la marche. Une problématique similaire se pose donc pour l'ensemble des maladies neuromusculaires à début pédiatrique se traduisant par une perte ou une absence d'acquisition de la marche.

A l'issue du workshop Treat-NMD du 11 juillet 2007 [3], le besoin de mesures spécifiquement adaptées pour évaluer la force résiduelle aux membres supérieurs de patients non ambulatoires a été soulevé. En effet, le test de six minutes de marche (4) qui a été proposé pour l'évaluation fonctionnelle de ces patients n'est évidemment pas adapté pour un patient non-ambulatoire. Dès lors, différentes échelles ont été proposées, parmi lesquelles le Hammersmith Functional Motor Scale (HFMS) (5), et la Mesure de Fonction Motrice (MFM) (6, 7). Ces différentes échelles ont comme avantage d'évaluer de manière complète les capacités motrices des membres supérieurs et ce, avec un minimum de matériel. Néanmoins, elles présentent comme inconvénient le temps nécessaire à leur réalisation et la dimension subjective de la mesure. La mesure quantifiée de la force de la main a récemment été proposée comme paramètre de l'évolution de la maladie pour des patients DMD (8), mais avec une mise en garde sur l'utilisation avant l'âge de 10 ans en raison d'une interférence possible avec la croissance.

Comme nous l'avons mentionné, il est indispensable de définir un test clinique simple, fiable et reproductible de la fonction musculaire pour évaluer l'effet de nouvelles approches thérapeutiques chez des patients non ambulatoires atteints de maladies neuromusculaires. A ce jour, nous ne disposons pas de test de ce type. Aussi, il est nécessaire de valider un outil dans ce cadre. Pour cela, nous avons besoin de pouvoir comparer ce test à d'autres tests déjà existants tels que des mesures de force musculaire, une échelle fonctionnelle (Mesure de la Fonction Motrice), et un auto-questionnaire (Indice fonctionnel de la main)

2. Objectifs

ULENAP est une étude de validation. Elle consiste à établir la pertinence d'un test clinique utilisé chez des patients non ambulatoires atteints de maladies neuromusculaires.

Cette étude a pour objet de développer un test clinique simple, fiable, reproductible, sensible et adapté aux patients non ambulatoires atteints de pathologie neuromusculaire (DMD, SMA, sarcoglycanopathie,…).

3. Méthodologie

Nous prévoyons d'inclure un groupe de 100 patients non ambulatoires atteints de maladies neuromusculaires, et 60 sujets contrôles uniformément répartis en sexe et en âge entre 8 et 30 ans. Les patients suivis à la consultation de pathologie neuromusculaire de l'Institut de Myologie à Paris, dans le service de Pédiatrie de l'Hôpital Universitaire Raymond Poincaré à Garches, dans le service de Neuropédiatrie de l'Hôpital Armand Trousseau à Paris et dans le service de Neuropédiatrie de l'Hôpital Necker-Enfants Malades à Paris pourront être inclus. Ils seront sélectionnés par le Docteur SERVAIS à l'Institut de Myologie, le Professeur Estournet et le Docteur Quijano-Roy à l'Hôpital Raymond Poincaré, le Docteur Mayer à l'Hôpital Armand Trousseau et le Docteur Desguerre à l'Hôpital Necker-Enfants Malades.

Les patients seront évalués dans le service où ils sont habituellement suivis, au cours d'une hospitalisation de jour ou à l'occasion d'une consultation. Le matériel d'évaluation sera dupliqué et fourni au service de pédiatrie de l'hôpital Universitaire Raymond Poincaré à Garches. Pour les autres centres, les évaluateurs de l'institut se déplaceront avec le matériel afin d'évaluer les patients. Une séance de formation kinésithérapeutes de Raymond Poincaré sera prévue avant les premières évaluations dans cet hôpital, et les premières évaluations seront réalisées en présence d'un évaluateur de l'Institut de myologie.

La reproductibilité intra- et inter-évaluateur (2 groupes de 20 patients et 2 groupes de 20 témoins) sera étudiée grâce à des mesures réalisées lors d'une deuxième évaluation soit le jour même de l'inclusion soit dans les 30 jours suivants.

Pour la sensibilité au changement, c'est-à-dire la capacité du test à détecter une détérioration attendue chez les patients, une évaluation à 6 mois devrait permettre de montrer que le Moviplate est plus sensible que les autres tests. Il est prévu également de tester à nouveau les témoins à 6 mois pour évaluer l'effet éventuel de l'âge (pour ceux en période de croissance). Dans l'éventualité où la sensibilité au changement n'aurait pas été démontrée à 6 mois, une visite à 12 mois est prévue pour tous les patients (ce rythme de suivi est celui habituellement proposé aux patients). Ces visites de suivi seront proposées aux patients présentant l'un des diagnostics les plus représentés (DMD, SMA, sarcoglycannopathie) afin de disposer d'un nombre suffisant de patients pour être analysés en sous-groupes.

4. Critères d'inclusion et d'exclusion des patients

Les patients non ambulatoires, suivis par les docteurs Servais, Estournet, Quijano-Roy, Mayer et Desguerre seront sollicités pour participer à cette étude. Une information directe au cours d'une consultation programmée leur sera donnée par le médecin en charge de leur suivi. Après ces explications orales, la lecture de la fiche d'information et la signature de leur consentement éclairé, les patients pourront être inclus dans l'étude.

4.1. Les critères d'inclusion

Pour les patients:

- Diagnostic confirmé par la génétique
- Age entre 8 et 30 ans
- Patient capable de tenir assis au fauteuil pendant au moins une heure
- Patients incapables de marcher 10 mètres dans les conditions standardisées du test de Six Minutes de marche [4]

- patient affilié à un régime de sécurité sociale
- Consentement éclairé signé

Nous prévoyons 100 patients.

Pour les sujets contrôles :

- 60 sujets contrôles uniformément répartis en âge et en sexe entre 8 et 30 ans. Ces contrôles seront recrutés sur le mode du volontariat
- Sujet affilié à un régime de sécurité sociale
- Consentement éclairé signé

<u>4.2. Les critères d'exclusion</u>

Pour les patients

- Patient incapable de tenir assis au fauteuil pendant au moins une heure
- Patients avec troubles cognitifs trop importants, limitant la compréhension des exercices à réaliser
- Patients ambulatoires
- Patients ayant subi une intervention chirurgicale ou ayant subi un traumatisme récent au niveau des membres supérieurs (moins de 6 mois),
- Patient sous corticoïdes depuis moins de six mois

Pour les contrôles

- Intervention chirurgicale ou traumatisme récent au niveau des membres supérieurs (moins de 6 mois),
- Sportif de haut niveau (niveau national),
- Traitement chronique altérant la force musculaire dans le mois précédant l'inclusion.

5. Calendrier et description des évaluations

Les patients et les sujets contrôles réaliseront le test Moviplate ainsi que d'autres tests qui permettront de vérifier la pertinence du test Moviplate (Tableau 1). Ces tests complémentaires sont les suivants :

-Mesures dynamométriques de force musculaire au niveau du poignet (extension) et de la main (préhension palmaire) (Annexe 2), des doigts (pince pouce-index) (Annexe 3)

-Test de Taping (Annexe 4)

-Echelle fonctionnelle : Mesure de la Fonction Motrice (Annexe 5) (pour les patients uniquement)

- Auto-questionnaire (Indice fonctionnel de la main) (Annexe 6)

L'ensemble des tests sera réalisé pour les 2 cotés du membre supérieur des sujets.

Pour les patients, les tests prendront environ 60 minutes et seront effectués à l'Institut de myologie, à l'Hôpital Raymond Poincaré, à l'Hôpital Armand Trousseau et à l'Hôpital Necker. Pour les sujets contrôles, les tests prendront environ 30 minutes et seront effectués à l'Institut de Myologie après obtention de leur consentement. Le jour même, après un temps de repos d'au minimum une heure, ou au plus tard 4 semaines après la première visite, une seconde évaluation sera effectuée avec le test Moviplate, les tests de force musculaires (poignet, main, doigts) et le test de taping (durée des tests estimée à 30 minutes).

A 6 mois : les patients (faisant partie des principales classes diagnostiques, DMD, SMA et sarcoglycannopathie) et les témoins auront une visite de suivi des évènements intervenus depuis l'inclusion dans l'étude, et les mêmes tests qu'à l'inclusion (voir tableau 1)

Tableau 1 : Calendrier des évaluations

	**Patients**				**Sujets contrôles**		
	**J0**	**<J30***	**J6mois**	**J12 mois**	**J0**	**<J30***	**J6mois**
**ANTECEDENTS MEDICO-CHIRURGICAUX**	X	X	X	X	X	X	X
**Moviplate**	X	X	X	X	X	X	X
**FORCE MUSCULAIRE :**							
- Extension poignet	X	X	X	X	X	X	X
- Préhension palmaire	X	X	X	X	X	X	X
- Pince pouce-index	X	X	X	X	X	X	X
**TAPING**	X	X	X	X	X	X	X
**MFM**	X		X	X			
**Autoquestionnaire (main)**	X		X	X	X		X
**Evts INTERCURRENTS**		X	X	X		X	X
**POIDS, TAILLE, FC, PA**	X	X	X	X	X	X	X

* le jour même ou les jours suivants dans la limite de 30 jours après l'inclusion.

Pour les patients, l'ensemble des tests sera à nouveau répété 12 mois après la première visite.

- Le test Moviplate (Annexe 1)

Ce test sera réalisé en position assise devant une table sur laquelle sera disposé l'outil Moviplate (Annexe 1). Cet outil est composé d'un plateau avec 2 petites plateformes surélevées que les sujets doivent toucher alternativement. Les sujets réalisent le test en position assise, leur avant-bras est fixé, ou non, sur le plateau positionné devant eux sur une table réglée à bonne hauteur. Le but de l'exercice est de faire un maximum d'aller-retour en 30 secondes entre les deux plateformes, grâce à un mouvement coordonné d'extension du poignet et des doigts. Le test est répété 2 fois à chaque évaluation (coté non dominant, coté dominant, puis coté non dominant, coté dominant).

- Mesures dynamométriques de force musculaire

Les mesures dynamométriques de la force maximale des fonctions suivantes seront effectuées : extension du poignet (outils Myotools) (Annexe 2), préhension palmaire (outils Myotools) (Annexe 2) et pince pouce-index (pinch) (Annexe 3).

- *Taping (Annexe 4)*

Le test consiste à taper sur un capteur de force avec l'index le plus de fois possible en 15 secondes. L'énergie utilisée, la fréquence, le nombre, l'intensité et la durée des contacts sont étudiés au cours du temps.

- *Mesure de la Fonction Motrice (6, 7) (Annexe 5)*

Cette échelle donne une mesure chiffrée des capacités motrices du patient. Elle comprend 32 items, certains statiques, d'autres dynamiques. Les items sont testés en position couchée, assise, debout et sont répartis en trois dimensions et cotés sur une échelle à 4 points (0=test non réalisé, 3=test réalisé complètement):

-D1 : station debout et transferts (13 items)

-D2 : motricité axiale et proximale (12 items)

-D3 : motricité distale (7 items)

- *Auto-questionnaire (Indice fonctionnel de la main)(9- 10) (Annexe 6)*

6. Recueil des données 1

Les résultats des mesures sont recueillis sur une fiche expérimentale donnée en Annexe 7.

L'ensemble des tests est non invasif et n'expose à aucun effet secondaire sérieux. Les risques associés à cette étude sont ceux liés à la réalisation de mouvements forcés (fatigue, douleurs musculaires, crampes, traumatismes musculaires ou musculo-tendineux liés à l'étirement). Les évaluations musculaires seront arrêtées en cas d'apparition d'une douleur ou d'une crampe.

7. Bénéfice attendu

Il n'y a pas de bénéfice attendu pour les patients se prêtant à ce protocole.

8. Durée d'exclusion

Il n'est pas prévu de période d'exclusion après l'inclusion des contrôles et des patients. Il n'y a pas de suivi prolongé, ni d'effets rémanents à prévoir.

9. Gestion des sorties d'essai

Les sujets qui ne pourront pas effectuer les mesures dans les conditions décrites dans le protocole ou qui ne voudront pas aller au bout du protocole pour une raison ou une autre seront remplacés par une autre personne d'âge similaire.

10. Gestion des événements indésirables

Il n'y a pas d'évènements indésirables graves prévisibles pour cette étude. Comme déjà souligné, il est précisé que les équipements utilisés ne sont que des matériels de quantification qui ne développent en eux-mêmes aucunes forces mais restent passivement résistants selon une force égale à la force développée par le sujet. Il n'existe aucun risque inhérent à l'utilisation des matériels (aucune mesure invasive, pas de contacts électriques). Seront relevés tous les événements indésirables, c'est-à-dire tous les symptômes, signes cliniques, réactions qui se développent ou s'aggravent lors de l'étude.

L'information sur tous les événements indésirables survenus sera immédiatement enregistrée à la place prévue à cet effet sur la fiche expérimentale et sera diffusée à l'investigateur principal ainsi qu'au promoteur. L'évolution de chaque événement sera suivie jusqu'à guérison, stabilisation ou jusqu'à ce qu'il soit clairement établi d'un commun accord que l'événement n'est pas lié à la recherche en cours.

11. Engagements de l'investigateur

La recherche sera menée dans le respect de la réglementation française en vigueur, notamment des dispositions relatives à la recherche biomédicale du Code le la Santé Publique, article L 1121-1 et suivants, des lois de Bioéthique, de la loi Informatique et Libertés, de la déclaration d'Helsinki, ainsi que des Recommandations de Bonne Pratique et du présent protocole.

L'investigateur s'engage à mener la recherche conformément à ces dispositions éthiques et réglementaires. Il est conscient que tous les documents ainsi que toutes les données relatives à la recherche pourront faire l'objet d'audits et d'inspections réalisées dans le respect du secret professionnel et sans que puisse être opposé le secret médical. L'investigateur reconnaît que les résultats de la recherche sont la propriété de l'Institut de Myologie, promoteur de la recherche.

Avant la mise en œuvre de la recherche, l'investigateur coordonnateur soumettra le projet à l'avis du CPP Ile-de-France VI (GH Pitié-Salpêtrière - 47, Boulevard de l'Hôpital - 75651 PARIS Cedex 13) en accord avec l'article L 209 de la loi Huriet de 1988 et lui fournira pour cela tous les renseignements nécessaires, formulaire d'information et de consentement ou tout autre document pertinent devant être présenté au Comité. L'essai ne pourra débuter que lorsque l'Institut de Myologie aura été informé de

l'avis favorable sans réserve délivré par le CPP à propos du protocole soumis. Le promoteur informera le CPP de tous les évènements indésirables graves ou inattendus et faits nouveaux apparaissant au cours de la recherche et qui affecteraient vraisemblablement la sécurité des personnes s'y prêtant.

L'Institut de Myologie, en tant que promoteur, a souscrit pour toute la durée de l'essai un contrat d'assurance sous le N°……………, conformément aux dispositions légales et réglementaires françaises sur les recherches biomédicales.

12. Lettre d'information et Consentement éclairé

Le consentement éclairé, écrit, des personnes qui se prêtent à la recherche, doit être obtenu par l'investigateur, inscrit à l'ordre des médecins, déclaré en tant qu'investigateur auprès du promoteur, avant tout acte pratiqué dans le cadre du protocole de la recherche et quel que soit cet acte, conformément à la réglementation.

Conformément à la loi du 20 décembre 1988 modifiée, l'information donnée oralement et par écrit aux patients comportera des renseignements sur l'objectif de la recherche, sa méthodologie et sa durée, les contraintes et les risques prévisibles, l'avis du CCP, ainsi que sur le droit de refuser de participer à la recherche ou de retirer son consentement à tout moment sans encourir aucune responsabilité. Les patients donneront leur consentement libre, éclairé et écrit en apposant leur signature et la date sur le formulaire de consentement. Ce formulaire de consentement sera rédigé en 2 exemplaires afin que le patient et le médecin-investigateur puissent en garder chacun un exemplaire.

L'investigateur devra s'assurer que la personne qui se prête à la recherche aura eu le temps de prendre sa décision librement et aura pu lire et comprendre le formulaire d'information et de consentement.

Le formulaire d'information et de consentement est un document qui aura été approuvé préalablement à la mise en œuvre de la recherche par le CPP, à l'occasion de l'examen du protocole.

Il n'est pas prévu d'indemniser les patients dans le cadre de ce protocole, à l'exception du remboursement des frais de déplacement.

13. La gestion des données

13.1. Confidentialité des données

Les données individuelles resteront confidentielles : la fiche de recueil des données sera référencée par un code en 4 chiffres (N° centre et N° d'inclusion de patient)

13.2 Analyse des données

La reproductibilité des tests sera estimée par analyse de coefficient de corrélation intra-classe (ICC) et par analyse de Bland et Altman. La sensibilité au changement sera évaluée par calcul des différences entre les évaluations de l'inclusion et celle de 6 et 12 mois. Pour comparer la sensibilité au changement du Moviplate à celle des autres tests, les Standardized Response Means (SRM) seront calculées par groupe diagnostic en regroupant les principaux (DMD, SMA, sarcoglycannopathie) et comparés à celle des témoins. Les valeurs de SRM values sont interprétées en utilisant la règle de suivante (11) : sensibilité faible (0.2 to 0.49), modérée (0.50 to 0.79), et importante (≥0.80).
Les corrélations entre les résultats du Moviplate et les autres tests. seront analysées pour la validité convergente.
L'évolution des mesures à 6 mois sera comparée entre les groupes patients et témoins par analyse de variance pour mesures répétées. Les effets de l'âge, sexe, poids (et autres données morphologiques) seront étudiés.

13.3 Archivage des documents

Les investigateurs conserveront un dossier comportant la liste des sujets inclus avec le code correspondant, la copie de tous les feuillets d'expérimentation et l'ensemble de la documentation relative à l'étude conformément à la réglementation européenne, pour une durée de 15 ans après la fin de l'étude.

Après saisie informatique des données, les originaux des documents seront conservés par l'investigateur.

14. Amendements au protocole

Ni les investigateurs, ni le promoteur, ne modifieront le protocole sans l'autorisation de l'autre partie. Toute modification substantielle du protocole, (par exemple : changement dans le déroulement de l'étude, extension de la durée de l'étude...) devra faire l'objet d'un amendement signé par l'investigateur principal et le promoteur. Il sera soumis pour avis au CPP. L'amendement dûment signé et approuvé sera porté à la connaissance et transmis à l'ensemble des collaborateurs de la recherche.

15. Durée de l'étude

La durée totale de l’étude sera de 36 mois. La durée d’inclusion des sujets sera d’environ 24 mois (entre septembre 2009 et septembre 2012) dès les accords obtenus. Pour rappel, les sujets sont inclus dans le protocole pour une durée d’environ 30 à 60 minutes par journée d’évaluation.

16. Rapport final et publications

A la fin de l’étude, un rapport sera rédigé par l’investigateur principal avec la collaboration des différents intervenants. Il pourra donner lieu à des communications écrites, orales ou affichées. Conformément au code de la Santé Publique, chacun des investigateurs ou intervenants s’interdit de publier ou de rapporter les résultats de l’étude sans l’accord formel des autres et information au promoteur.

17. Bibliographie

1. Engel AG & Ozawa E. Dystrophinopathies, In Myology, third ed., Engel AG & Franzini-Armstrong C, Mc Graw-Hill, NY 2004. p 961-1026

2. Rudnick-Scöenberg S, de Visser M, Zeros K. Spinal Muscular Atrophies. In Myology, third ed., Engel AG & Franzini-Armstrong C, Mc Graw-Hill, NY 2004. p 1845-1864.

3. Mercuri, E., Mayhew, A., Muntoni, F., Messina, S., Straub, V., Van Ommen, G.J., Voit, T., Bertini, E., Bushby, K. (2008). Towards harmonisation of outcome measures for DMD and SMA within TREAT-NMD; report of three expert workshops: TREAT-NMD/ENMC workshop on outcome measures, 12th--13th May 2007, Naarden, The Netherlands; TREAT-NMD workshop on outcome measures in experimental trials for DMD, 30th June--1st July 2007, Naarden, The Netherlands; conjoint Institute of Myology TREAT-NMD meeting on physical activity monitoring in neuromuscular disorders, 11th July 2007, Paris, France. *Neuromuscul. Disord. 18,* 894-903.

4. ATS statement: guidelines for the six-minute walk test. Am J Respir Crit Care Med 2002; 166 (1): 111-117.

5. Main, M., Kairon, H., Mercuri, E. and Muntoni, F. (2003). The Hammersmith functional motor scale for children with spinal muscular atrophy: a scale to test ability and monitor progress in children with limited ambulation. *Eur. J. Paediatr. Neurol. 7*, 155-159.

6. Bérard, C., Payan, C., Hodgkinson, I., Fermanian, J. (2005). A motor function measure for neuromuscular diseases. Construction and validation study. *Neuromuscul. Disord.15*, 463-470.

7. Bérard, C., Payan, C., Fermanian, J., Girardot, F. (2006). [A motor function measurement scale for neuromuscular diseases - description and validation study]. *Rev. Neurol. (Paris) 162*, 485-493.

8. Mattar, F.L. and Sobreira, C. (2008). Hand weakness in Duchenne muscular dystrophy and its relation to physical disability. *Neuromuscul. Disord. 18*, 193-198.

9. Sezer N, Yavuzer G, Sivrioglu K,Basaran P, Koseoglu F. (2007). Clinimetric properties of the Duruoz Hand Index in patient with stroke. *Arch. Phys. Med. Rehabil.* 88 : 309-314.

10. Poireaudeau S, Lefèvre-Colau MM, Fermanian J, Mayoux-Benhamou MA, Revel M. (2000). Qualités métrologiques de l'indice d'incapacité fonctionnelle de Cochin adapté à la main rhumatoïde. *Ann. Réadaptation Med Phys.* 43 : 106-115.

11 . Bjorgvinsson T, Kerr P. (1995) Use of a common language effect size statistic. Am. J. Psychiatry 152:151.

18. Annexes

Annexe 1 : Outil ***Moviplate***

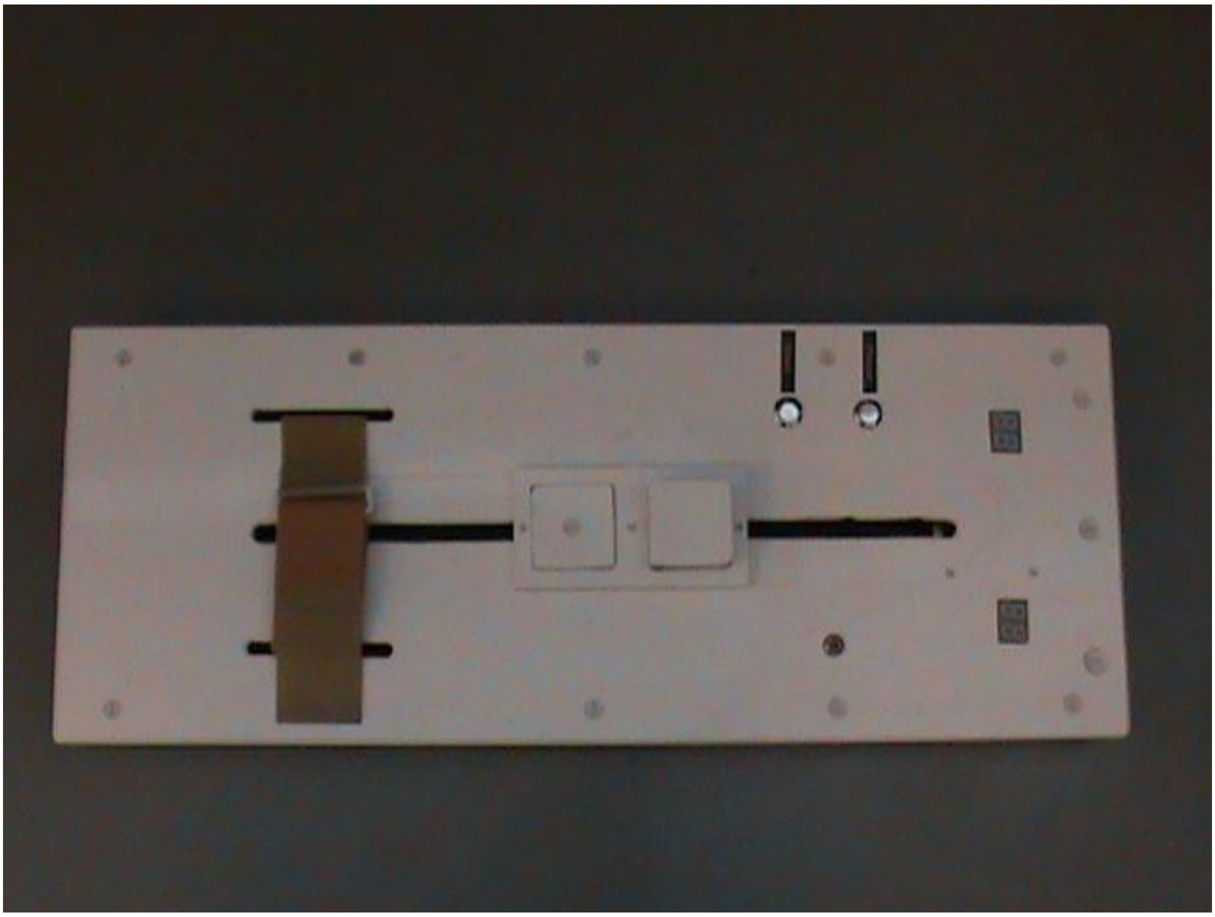

a : Chronomètre. En seconde, option compteur et décompteur, écran visualisable ou non.

b : Compteur. En valeur absolue, décompte visualisable. Il compte le nombre de contact avec c

c : Plateforme d’enregistrement, réglable en hauteur (1 ou 2 cm de hauteur), et ajustable sur la longueur de la planche (cf schéma). Elle permet d’enregistrer sans analyse préalable la valeur discrète constituée par le nombre de contacts avec c pendant la durée du chronométrage.

d : Pieds

e : Fente pour ajustement de lanière de contention de l’avant bras.

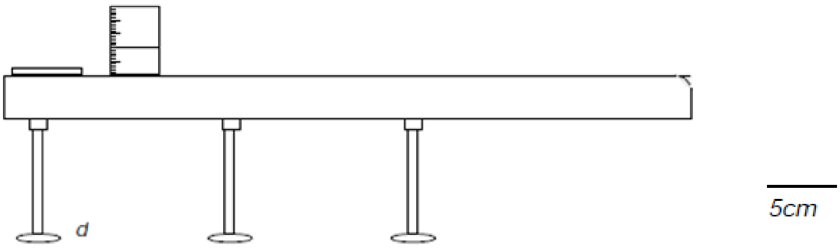

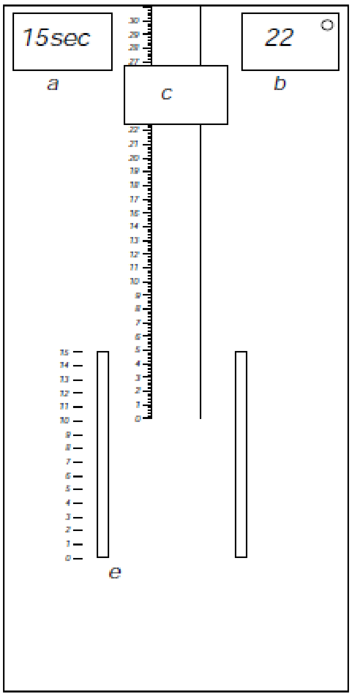

Annexe 2: outils Myotools

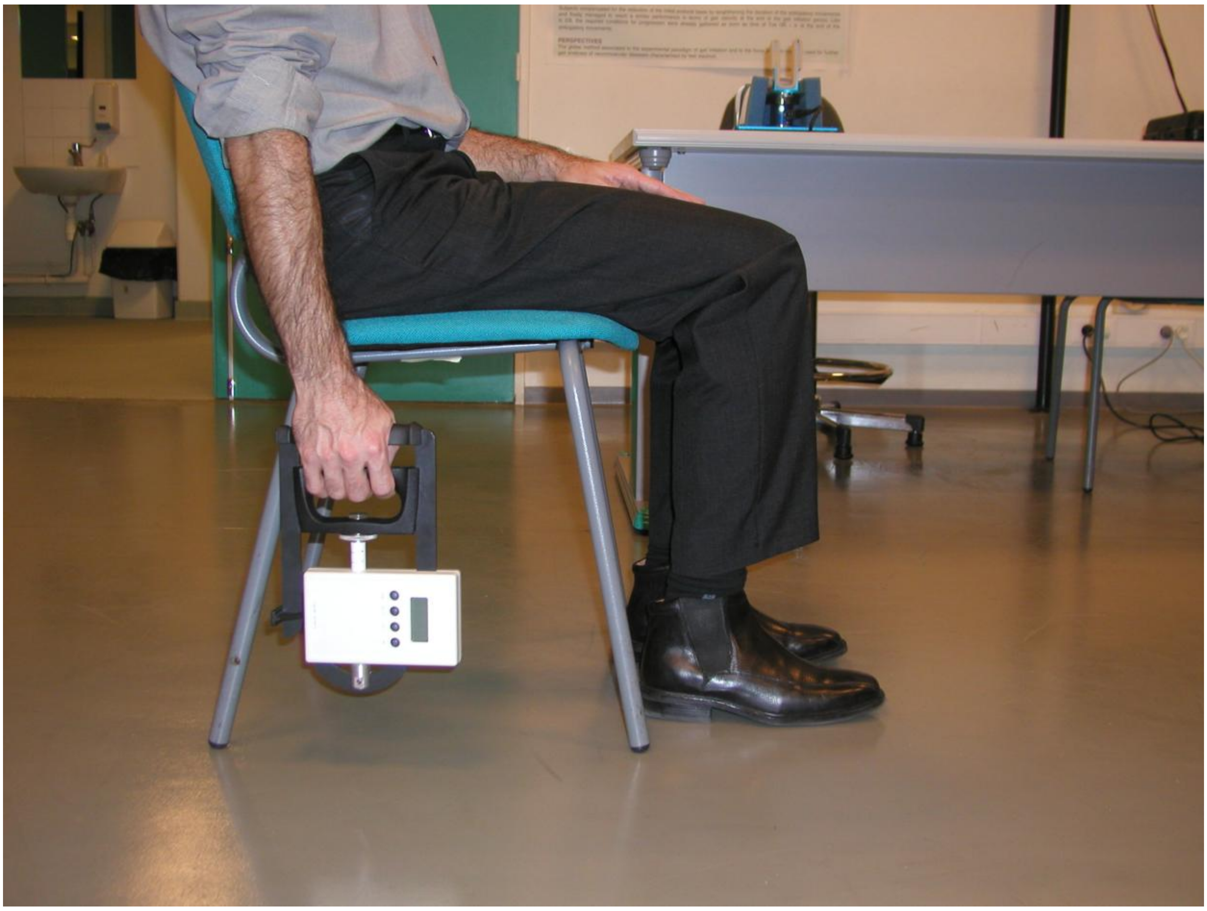

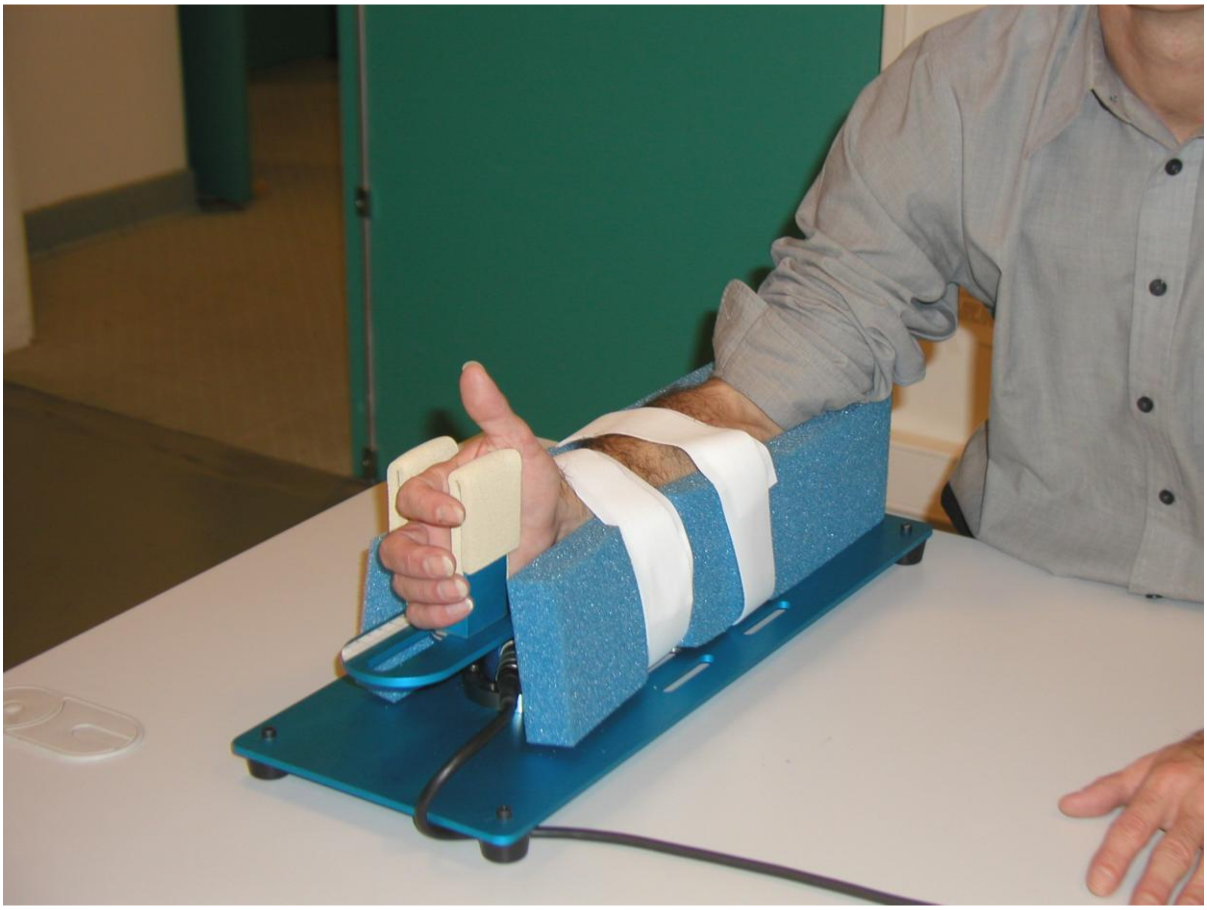

Annexe 3: Pinch

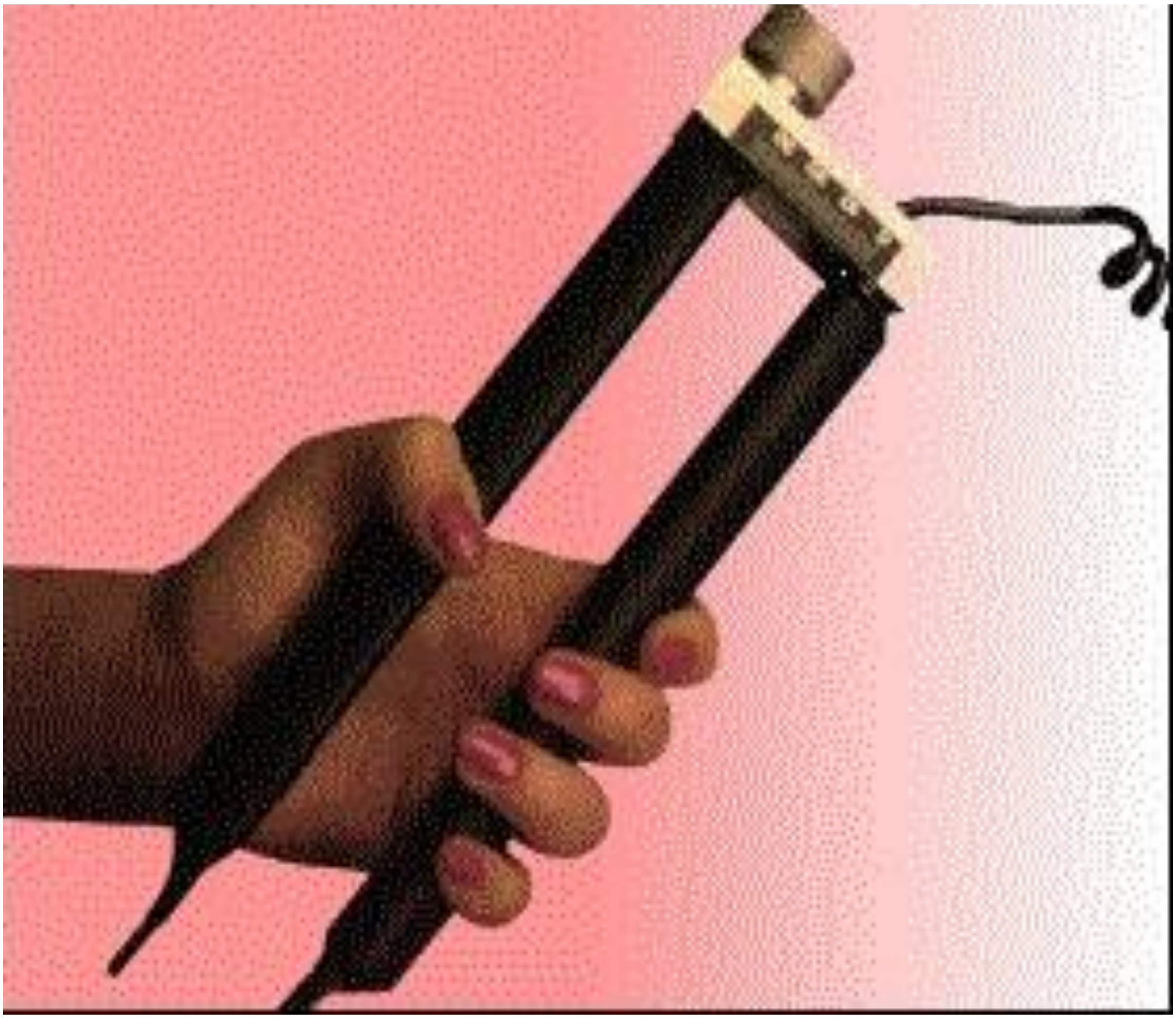

Annexe 4: Taping

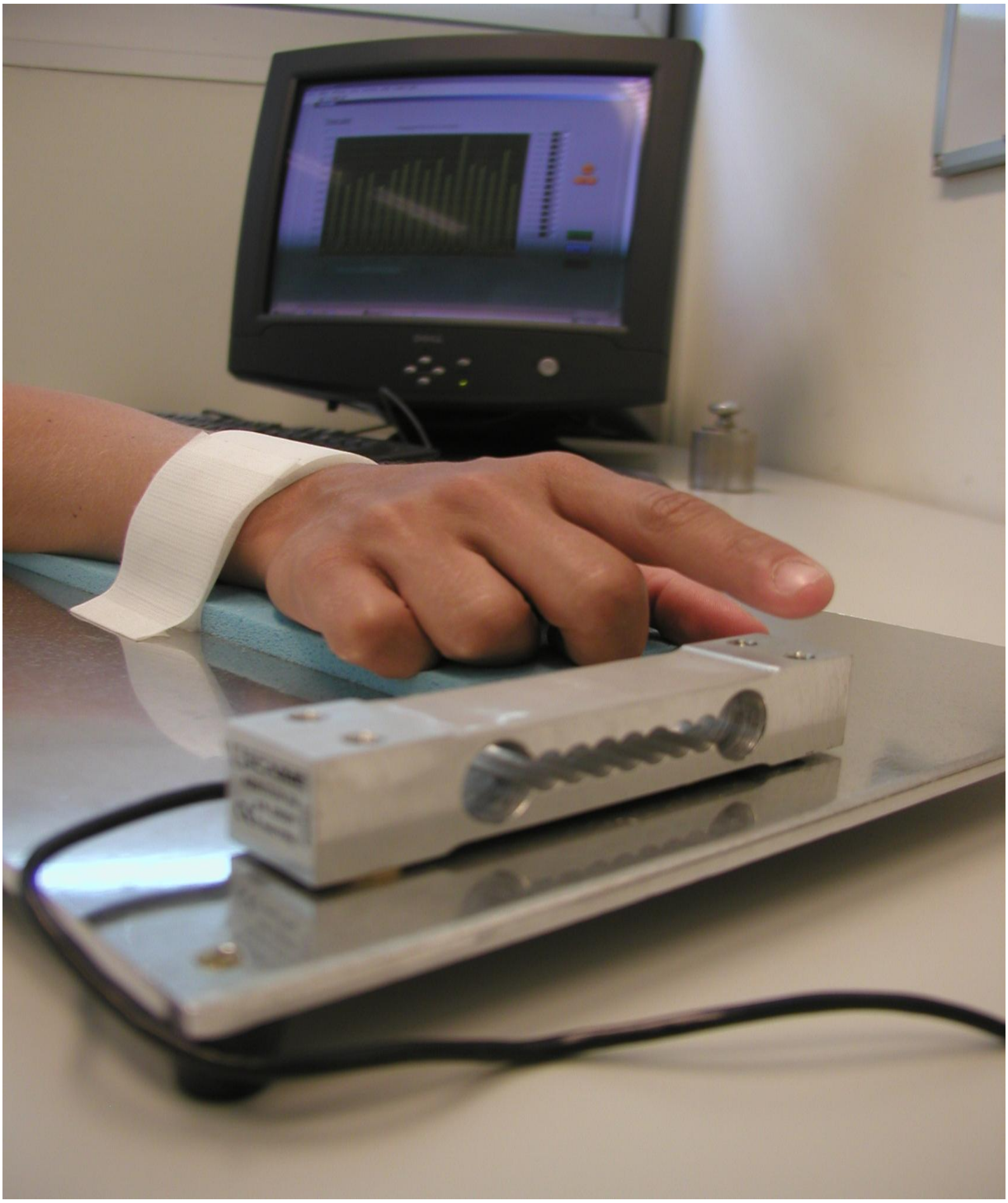

Annexe 5 : Echelle de Mesure de Fonction Motrice (MFM)

Cotation

**Items de l'échelle MFM**	**D1 =**	**D2 =**	**D3 =**
1. Couché sur le dos, tête dans l'axe : maintient la tête dans l'axe 5 secondes puis la tourne complètement d'un côté puis de l'autre<br>*commentaires* :		□ 0<br>□ 1<br>□ 2<br>□ 3	
2. Couché sur le dos : soulève la tête et la maintient soulevée 5 secondes.<br>*commentaires* :		□ 0<br>□ 1<br>□ 2<br>□ 3	
3. Couché sur le dos : fléchit la hanche et le genou de plus de 90° en soulevant le pied durant tout le mouvement.<br>côté choisi : droit 1 : □<br>gauche 2 : □<br>cotation controlatérale : □0 □1 □2 □3<br>*commentaires* :		□ 0<br>□ 1<br>□ 2<br>□ 3	
4. Couché sur le dos, jambe soutenue par l'examinateur : de la position pied en flexion plantaire, réalise une flexion dorsale du pied à au moins 90° par rapport à la jambe.<br>côté choisi : droit 1 : □<br>gauche 2 : □<br>cotation controlatérale : □0 □1 □2 □3<br>*commentaires* :			□ 0<br>□ 1<br>□ 2<br>□ 3
5. Couché sur le dos : soulève la main et la porte jusqu'à l'épaule opposée.<br>côté choisi : droit 1 : □<br>gauche 2 : □<br>cotation controlatérale : □0 □1 □2 □3<br>*commentaires* :		□ 0<br>□ 1<br>□ 2<br>□ 3	
6. Couché sur le dos, membres inférieurs demi fléchis, rotules au zénith, pieds sur le tapis légèrement écartés: maintient 5 secondes la position de départ puis soulève le bassin ; la colonne lombaire, le bassin et les cuisses sont alignés et les pieds légèrement écartés.<br>*commentaires* :	□ 0<br>□ 1<br>□ 2<br>□ 3		

Items de l'échelle MFM	D1 =	D2 =	D3 =
7. Couché sur le dos : se retourne sur le ventre et dégage les membres supérieurs de dessous le tronc.<br>côté choisi : droit 1 : ☐ gauche 2 : ☐<br>cotation controlatérale : ☐0 ☐1 ☐2 ☐3<br>*commentaires* :		☐ 0<br>☐ 1<br>☐ 2<br>☐ 3	
8. Couché sur le dos : sans appui des membres supérieurs, s'assied.<br>*commentaires* :	☐ 0<br>☐ 1<br>☐ 2<br>☐ 3		
9. Assis sur le tapis : sans appui des membres supérieurs, maintient 5 secondes la station assise puis maintient 5 secondes un contact entre les 2 mains.<br>*commentaires* :		☐ 0<br>☐ 1<br>☐ 2<br>☐ 3	
10. Assis sur le tapis, la balle de tennis devant le sujet : sans appui des membres supérieurs se penche en avant, touche la balle puis se redresse.<br>..<br>..<br>*commentaires* :		☐ 0<br>☐ 1<br>☐ 2<br>☐ 3	
11. Assis sur le tapis : sans appui des membres supérieurs, se met debout.<br>*commentaires* :	☐ 0<br>☐ 1<br>☐ 2<br>☐ 3		
12. Debout : sans appui des membres supérieurs, s'assied sur la chaise en gardant les pieds légèrement écartés.<br>*commentaires* :	☐ 0<br>☐ 1<br>☐ 2<br>☐ 3		
13. Assis sur la chaise : sans appui des membres supérieurs et sans appui contre le dossier de la chaise, maintient 5 secondes la position assise, tête et tronc dans l'axe.<br>*commentaires* :		☐ 0<br>☐ 1<br>☐ 2<br>☐ 3	
14. Assis sur la chaise ou dans son fauteuil, tête positionnée en flexion : de la position tête fléchie complètement, relève la tête puis la maintient relevée 5 secondes, le mouvement et le maintien se faisant tête dans l'axe.<br>*commentaires* :		☐ 0<br>☐ 1<br>☐ 2<br>☐ 3	
15. Assis sur la chaise ou dans son fauteuil, avant-bras posés sur la table, coudes en dehors de la table : porte en même temps les 2 mains sur le sommet du crâne, la tête et le tronc restant dans l'axe.<br>*commentaires* :		☐ 0<br>☐ 1<br>☐ 2<br>☐ 3	

**Items de l'échelle MFM**	**D1 =**	**D2 =**	**D3 =**
16. Assis sur la chaise ou dans son fauteuil, le crayon sur la table : sans bouger le tronc, atteint le crayon avec la main, avant-bras et main soulevés de la table, coude en extension complète en fin de mouvement.<br>côté choisi : droit 1 : ☐ gauche 2 : ☐<br>cotation controlatérale : ☐0 ☐1 ☐2 ☐3<br>*commentaires* :		☐ 0<br>☐ 1<br>☐ 2<br>☐ 3	
17. Assis sur la chaise ou dans son fauteuil, 10 pièces de monnaie sur la table : prend successivement et stocke 10 pièces dans une main au bout de 20 secondes.<br>côté choisi : droit 1 : ☐ gauche 2 : ☐<br>cotation controlatérale : ☐0 ☐1 ☐2 ☐3<br>*commentaires* :			☐ 0<br>☐ 1<br>☐ 2<br>☐ 3
18. Assis sur la chaise ou dans son fauteuil, un doigt posé au centre d'un CD fixe : fait le tour du CD avec le même doigt, sans appui de la main.<br>côté choisi : droit 1 : ☐ gauche 2 : ☐<br>cotation controlatérale : ☐0 ☐1 ☐2 ☐3<br>*commentaires* :			☐ 0<br>☐ 1<br>☐ 2<br>☐ 3
19. Assis sur la chaise ou dans son fauteuil, le crayon sur la table : prend le crayon puis dessine une série continue de boucles sur toute la longueur du cadre touchant les bords supérieur et inférieur du cadre.<br>côté choisi : droit 1 : ☐ gauche 2 : ☐<br>cotation controlatérale : ☐0 ☐1 ☐2 ☐3<br>*commentaires* : ....<br>**Essai n°1** essai controlatéral n°1<br>**Essai n°2** essai controlatéral n°2			☐ 0<br>☐ 1<br>☐ 2<br>☐ 3
20. Assis sur la chaise ou dans son fauteuil, la feuille de papier dans les mains : déchire la feuille pliée en 4 en commençant par le pli.<br>*commentaires* :			☐ 0<br>☐ 1<br>☐ 2<br>☐ 3

Items de l'échelle MFM	D1 =	D2 =	D3 =
21. Assis sur la chaise ou dans son fauteuil, la balle de tennis sur la table : soulève la balle puis retourne la main complètement en tenant la balle.<br>côté choisi : droit 1 : □ gauche 2 : □<br>cotation controlatérale : □0 □1 □2 □3<br>*commentaires* :			□ 0<br>□ 1<br>□ 2<br>□ 3
22. Assis sur la chaise ou dans son fauteuil, un doigt posé au centre du carré: soulève le doigt puis le pose successivement dans les 8 carrés de la figure sans toucher le quadrillage.<br>côté choisi : droit 1 : □ gauche 2 : □<br>cotation controlatérale : □0 □1 □2 □3<br>*commentaires* :			□ 0<br>□ 1<br>□ 2<br>□ 3
23. Assis sur la chaise ou dans son fauteuil, membres supérieurs le long du corps : pose en même temps les 2 avant-bras et/ou les mains sur la table sans bouger le tronc.<br>*commentaires* :		□ 0<br>□ 1<br>□ 2<br>□ 3	
24. Assis sur la chaise : sans appui des membres supérieurs, se met debout les pieds légèrement écartés.<br>*commentaires* :	□ 0<br>□ 1<br>□ 2<br>□ 3		
25. Debout avec appui des membres supérieurs sur un matériel : sans appui des membres supérieurs, maintient 5 secondes la position pieds légèrement écartés, tête, tronc et membres inférieurs dans l'axe.<br>*commentaires* :	□ 0<br>□ 1<br>□ 2<br>□ 3		
26. Debout avec appui des membres supérieurs sur un matériel : sans appui des membres supérieurs, lève un pied 10 secondes.<br>côté choisi : droit 1 : □ gauche 2 : □<br>cotation controlatérale : □0 □1 □2 □3<br>*commentaires* :	□ 0<br>□ 1<br>□ 2<br>□ 3		
27. Debout : sans appui, touche le sol avec une main puis se relève.<br>*commentaires* :	□ 0<br>□ 1<br>□ 2<br>□ 3		
28. Debout sans appui : fait 10 pas en avant sur les 2 talons.<br>*commentaires* :	□ 0<br>□ 1<br>□ 2<br>□ 3		
29. Debout sans appui : fait 10 pas en avant sur une ligne droite.<br>*commentaires* :	□ 0<br>□ 1<br>□ 2<br>□ 3		

**Items de l'échelle MFM**	**D1 =**	**D2 =**	**D3 =**
30. Debout sans appui : court 10 mètres.<br>*commentaires* :	☐ 0<br>☐ 1<br>☐ 2<br>☐ 3		
31. Debout sur un pied sans appui : saute sur un pied 10 fois de suite sur place.<br>côté choisi : droit 1 : ☐ gauche 2 : ☐<br>cotation controlatérale : ☐0 ☐1 ☐2 ☐3<br>*commentaires* :	☐ 0<br>☐ 1<br>☐ 2<br>☐ 3		
32. Debout sans appui : sans appui des membres supérieurs, atteint la position accroupie puis se relève 2 fois de suite.<br>*commentaires* :	☐ 0<br>☐ 1<br>☐ 2<br>☐ 3		
**TOTAL**	**D1=**	**D2=**	**D3=**

Durée de la passation : I___I___I___I Minutes

Coopération du patient : nulle I___I 0, moyenne I___I 1, optimale I___I 2

Commentaires sur le déroulement du test et sur les résultats obtenus: (*texte libre, si rien de particulier, noter RAS*)
………………………………………………………………………………………………………
………………………………………………………………………………………………………
……………………………………………...

Réponses aux questions : 0 = oui sans difficulté ; 1 = possible avec très peu de difficultés ; 2 = possible avec quelques difficultés ; 3 = possible avec beaucoup de difficultés ; 4 = presque impossible ; 5 = impossible.
Veuillez répondre aux questions ci-dessous, sans appareillage adapté :

C1 – A la cuisine
Pouvez-vous tenir un bol ?
Pouvez-vous saisir une bouteille pleine et la lever ?
Pouvez-vous tenir un plat plein ?
Pouvez-vous verser le liquide de la bouteille dans un verre ?
Pouvez-vous dévisser le couvercle d'un pot déjà ouvert une fois ?
Pouvez-vous couper la viande avec un couteau ?
Pouvez-vous piquer efficacement avec une fourchette ?
Pouvez-vous peler des fruits ?

C2 – Habillage
Pouvez-vous boutonner votre chemise
Pouvez vous ouvrir puis fermer les fermetures éclairs ?

C3 – Toilette
Pouvez-vous presser un tube de dentifrice avec un crayon ou un stylo ordinaire ?
Pouvez-vous tenir votre brosse à dent efficacement ?

C4 – Au bureau
Pouvez-vous écrire une phrase courte avec un crayon ou un stylo ordinaire ?
Pouvez-vous écrire une lettre avec un crayon ou un stylo ordinaire ?

C5 – Divers
Pouvez-vous tourner une poignet de porte ronde ?
Pouvez-vous utiliser des ciseaux pour couper un morceau de papier ?
Pouvez-vous saisir les pièces de monnaie sur une table ?
Pouvez-vous tourner une clé dans la serrure ?

Annexe 7 : Fiche expérimentale

Code sujet : |__|__|__| |__|__|__| |__|__|

Date de la visite : |__|__|-|__||__|-|__|__|__|__| Heure de la visite : |__|__|:|__|__|

Visite n° |__|

Date de naissance : |__|__|-|__||__|-|__|__|__|__|

Poids : |__|__|__|,|__| kg Taille : |__|__|__|,|__| cm %ge Masse grasse : |__|__|,|__|

Côté Dominant : Gauche ☐ Droit ☐ Ambidextre ☐

Circonférence avant-bras |__|__|,|__| cm Circonférence main |__|__|,|__| cm
Taille main |__|__|,|__| cm

Observations (antécédents traumatiques, douleurs, pathologies de l'appareil locomoteur...)
……………………………………………………………………………………………………
……………………………………………………………………………………………………
………………………………………………………………………..

Moviplate Evaluateur (…………..…….)

Position de la cible |__|__|,|__| cm
Hauteur de la cible 1cm☐ 2cm ☐

Avant-bras fixé :
Position de la sangle de fixation |__|__|,|__| cm

	1. heure |__|__|:|__|__|	2. heure |__|__|:|__|__|
Nb touchers de cible (30s)	G |__|__| D |__|__|	G |__|__| D |__|__|

Commentaires :
……………………………………………………………………………………………………
…………………………………………………………………………………………

Avant-bras libre :
Position de l’appui levier de l’avant-bras |__|__|,|__| cm de la pointe inférieure de la styloïde radiale

	1. heure |__|__|:|__|__|	2. heure |__|__|:|__|__|
Nb touchers de cible (30s)	G |__|__| D |__|__|	G |__|__| D |__|__|

Commentaires :
………
…………………………………………………………………………………………

Préhension palmaire (poignée IDM) Evaluateur (…..................…...)

Largeur poignée: |__|,|__|

		1	2	3	4	5
G	MVC (daN)	|__|__|,|__|__|	|__|__|,|__|__|	|__|__|,|__|__|	|__|__|,|__|__|	|__|__|,|__|__|
D	MVC (daN)	|__|__|,|__|__|	|__|__|,|__|__|	|__|__|,|__|__|	|__|__|,|__|__|	|__|__|,|__|__|

Commentaires :
………
…………………………………………………………………………………………

Extension de poignet (outil Myotools) Evaluateur (………………….…...)

Distance appui palmaire : |__|__|,|__| cm
Hauteur table : |__|__|,|__| cm

		1	2	3	4	5
G	MVC (N.m)	|__|__|,|__|__|	|__|__|,|__|__|	|__|__|,|__|__|	|__|__|,|__|__|	|__|__|,|__|__|
D	MVC (N.m)	|__|__|,|__|__|	|__|__|,|__|__|	|__|__|,|__|__|	|__|__|,|__|__|	|__|__|,|__|__|

Commentaires :
………
…………………………………………………………………………………………

Pince pouce-index (pinch) Evaluateur (……………....…...)

		1	2	3	4	5
G	MVC (kg)	|__|__|,|__|__|	|__|__|,|__|__|	|__|__|,|__|__|	|__|__|,|__|__|	|__|__|,|__|__|
D	MVC (kg)	|__|__|,|__|__|	|__|__|,|__|__|	|__|__|,|__|__|	|__|__|,|__|__|	|__|__|,|__|__|

Commentaires :
………
…………………………………………………………………………………………

Taping Evaluateur (……………....…...)

Réglages
Nombre de taps (15 secondes) |__|__|__|

Commentaires :
………
…………………………………………………………………………………………
