# Supplementary material for: Upper Limb Evaluation and One-Year Follow Up of Non-Ambulant Patients with Spinal Muscular Atrophy: An Observational Multicenter Trial
Source: PLoS One. 2015 Apr 10;10(4):e0121799. doi: 10.1371/journal.pone.0121799 (PMC4393256; doi:10.1371/journal.pone.0121799)
Supplement: S2 Protocol — (PDF) [file pone.0121799.s003.pdf]

PROJECT OF BIOMEDICAL RESEARCH

Development of a clinical test aimed to assess the capacity of upper limbs in non- ambulant patients with neuromuscular diseases.

ULENAP Study (Upper Limb Evaluation in Non-Ambulant Patients)

Promoter:

Association Institut de Myologie (AIM) - GH Pitié-Salpêtrière - 75651 PARIS Cedex 13

Principal investigator:

Dr Laurent Servais, Institut de Myologie, GH Pitié-Salpêtrière

Tel: 01 42 16 99 66

***Investigators*:**

Laurent Servais, Susana Quijano-Roy, Michèle Mayer, Isabelle Desguerre, Brigitte Estournet,

Collaborating scientists:

Jean-Yves Hogrel, Aurélie Canal

Experimental plan:

Multicenter study without direct personal profit

Places of the study:

- Center of Reference for Neuromuscular Pathologies – Paris Est, Institute of Myology - Pitié-Salpêtrière, Paris, France.

- Department of Pediatrics, Raymond Poincaré University Hospital, National Center of Reference for Neuromuscular Diseases Garches-Necker-Mondor-Hendaye, Garches, France.

- Department of Pediatric Neurology, AP-HP, Armand Trousseau Hospital, Paris, France.

- Department of Pediatric Neurology, AP-HP, the Necker-Enfants Malades Hospital, Paris, France.

- Laboratory of Neuromuscular Physiology and Neuromuscular Evaluation, Institute of Myology - Pitié-Salpêtrière, Paris, France.

AFSSAPS registration N°: 2009-A00600-57

SUMMARY

1. INTRODUCTION ... *2*

2. OBJECTIVES ... *4*

3. METHODOLOGY ... *4*

4. PATIENTS' INCLUSION AND EXCLUSION CRITERIA ... *5*

4.1. THE INCLUSION CRITERIA. ... 5
4.2. THE EXCLUSION CRITERIA ... 5

5. CALENDAR AND DESCRIPTION OF THE EVALUATIONS ... *6*

- THE MOVIPLATE TEST (APPENDIX 1) ... 7
- DYNAMOMETRICAL MEASUREMENTS OF MUSCULAR STRENGTH ... 7
- TAPING (APPENDIX 4) ... 8
- MOTOR FUNCTION MEASURE (6, 7) (APPENDIX 5) ... 8
- SELF-ADMINISTERED QUESTIONNAIRE (PERFORMANCE STATUS OF A HAND (9-10) (APPENDIX 6) ... 8

6. DATA COLLECTION ... *8*

7. EXPECTED PROFIT ... *8*

8. DURATION OF THE EXCLUSION ... *8*

9. MANAGEMENT OF WITHDRAWAL FROM A TRIAL ... *9*

10. MANAGEMENT OF ADVERSE EFFECTS ... *9*

11. INVESTIGATOR'S COMMITMENTS ... *9*

12. INFORMATION NOTICE AND INFORMED CONSENT ... *10*

13. DATA MANAGEMENT ... *10*

13.1. PRIVACY POLICY ... 10
13.2 DATA ANALYSIS ... 11

14. AMENDMENTS TO THE PROTOCOL ... *11*

15. DURATION OF THE STUDY ... *11*

16. FINAL REPORT AND PUBLICATIONS ... *12*

17. BIBLIOGRAPHIE ... *13*

18. ANNEXES ... *14*

ANNEXE 1 : OUTIL MOVIPLATE ... 14
ANNEXE 2: OUTILS MYOTOOLS ... 15
ANNEXE 3: PINCH ... 16
ANNEXE 4: TAPING ... 17
ANNEXE 5 : ECHELLE DE MESURE DE FONCTION MOTRICE (MFM) ... 18
ANNEXE 6 : AUTO-QUESTIONNAIRE (INDICE FONCTIONNEL DE LA MAIN) ... 23
ANNEXE 7 : FICHE EXPERIMENTALE ... 24

1. Introduction

Neuromuscular diseases constitute a group of heterogeneous pathologies in regards to their genetic origin, their symptoms, their natural history and their prognosis. Among them, some diseases begin in childhood or even in utero and lead to a loss or absence of acquisition of walking. The two most common diseases in this subgroup are Duchene Muscular Dystrophy (DMD) and Spinal Muscular Atrophy Type II (SMA II).

Duchene Muscular Dystrophy (DMD) is the most common children's myopathy (1). It affects all the muscles of the body. It appears only in boys before the age of five and leads to a loss of walking before the ages of ten and twelve. The use of upper limbs gradually decreases. The diagnosis is made due to a muscle biopsy and it is confirmed genetically with the identification of a deletion or a mutation in the dystrophin gene.

Spinal Muscular Atrophy Type II (SMA II) is connected with a mutation of the *SMN* gene causing the death of spinal motor neurons (2). Clinically, it is characterized by a loss of proximal and distal strength in children, girls and boys who have acquired sitting position but not walking.

Other less common diseases, such as the sarcoglycanopathies, alpha dystroglycanopathies, some myasthenia or congenital myopathies, the merosin negative dystrophy, Ullrich muscular dystrophy, even some forms of laminopathies can also appear in childhood and lead to a loss or absence of acquisition of walking. A similar problem arises, therefore, for all pediatric neuromuscular diseases leading to a loss or absence of acquisition of walking.

After the Treat-NMD workshop of 11 July 2007 (3), the need for specifically adapted measures to assess the residual strength of upper limbs in non- ambulant patients was raised.

Indeed, the six-minute walk test (4) which has been proposed for the functional evaluation of these patients is not obviously suitable for a non- ambulant patient. Therefore, different scales have been proposed, including the Hammersmith Functional Motor Scale (HFMS) (5), and Motor Function Measure (MFM) (6, 7). These scales have the advantage of full evaluation of motor skills and upper limbs, with a minimum of equipment. However, they have the disadvantage of the time required for their application and the subjective dimension of the measure. The quantified measure of the hand strength has recently been proposed as a parameter of disease progression for DMD patients (8), but with a special caution on using it before the age of ten due to a possible interference with the process of growth.

As we mentioned before, it is essential to define a simple, reliable and reproducible clinical test of muscle function to assess the effect of new therapeutic approaches for non- ambulant patients with neuromuscular diseases. These days we have no such a test. At the same time, in this context, a validation of such tool is needed. To do this, we need to compare this test with other already existing tests such as the muscular strength measurements, functional scales (Motor Function Measure) and self-administered questionnaire (Functional status of a hand).

2. Objectives

ULENAP is a validation study. It aims to establish the relevance of a clinical test used for non-ambulant patients with neuromuscular diseases.
The aim of the study is to develop a simple, reliable, reproducible, sensitive clinical test suitable for non-ambulant patients with neuromuscular diseases (DMD, SMA, sarcoglycanopathy, ...).

3. Methodology

We plan to include a group of one hundred non-ambulant patients with neuromuscular diseases and sixty control subjects uniformly distributed in regards to age and gender, with ages between eight and thirty. Patients are to have the neuromuscular consultation at the Institute of Myology in Paris, in the Pediatrics department of the Raymond Poincaré University Hospital in Garches, in the Pediatric Neurology department of the Armand Trousseau Hospital in Paris and in the Pediatric Neurology department of the Necker-Enfants Malades Hospital in Paris. They will be selected by Dr. SERVAIS at the Institute of Myology, Professor Estournet and Dr. Quijano-Roy at the Raymond Poincaré Hospital, Dr. Mayer at the Armand Trousseau Hospital and Dr. Desguerre at the Necker-Enfants Malades Hospital**.**
Patients will be assessed during a daily hospitalization or during a consultation in the department where they are usually followed-up. Evaluating materials will be duplicated and provided to the Pediatrics department of the Raymond Poincaré University Hospital in Garches. For other centers, the evaluators from the Institute will move with the material to assess the patients. A training session for physiotherapists of the Raymond Poincaré Hospital will be scheduled before the first evaluations at the hospital; and the first evaluations will be conducted in the presence of an evaluator of the Institute of Myology.
The intra and inter rater reliability **(**two groups of twenty patients and two groups of twenty controls) will be studied after a second evaluation, done the same day of the inclusion or within the next thirty days.
For the sensitivity to change, that is to say the ability of the test to detect an expected deterioration in patients, the evaluation in six months is expected to show that the Moviplate is more sensitive than other tests. It is also planned to test new controls in six months to assess the potential effect of age (for those in period of growth). In case if the change sensitivity is not demonstrated in six months, a visit for all the patients is planned in twelve months (this follow-up is usually offered to patients). These follow-up visits will be proposed to patients with the most represented diagnosis (DMD, SMA, sarcoglycannopathie) in order to have a sufficient number of patients to be analyzed in each subgroup.

4. Patients' inclusion and exclusion criteria

Non-ambulant patients, treated by doctors Servais, Estournet, Quijano-Roy, Mayer or Desguerre, will be called on to participate in this study. During a scheduled consultation, the doctor in charge will directly inform them. After the oral discussion, reading the information notice and signing the informed consent, the patients can be included in the study.

4.1. The inclusion criteria.

For the patients:

- Genetically confirmed diagnosis
- Ages between eight and thirty
- Patient able to sit on a chair for at least an hour.
- Patient to able to walk ten meters in the standardized conditions of the Six- Minute walk test [4]
- Patients assigned to a social security system.
- Signed informed consent

We expect to include a hundred patients.

For the control subjects:

- Sixty control subjects uniformly distributed by age and gender, with ages between eight and thirty. These controls will be recruited on a voluntary basis
- Patients assigned to a social security system. Signed informed consent

4.2. The exclusion criteria

For the patients

- Patient not able to sit on a chair for at least an hour.
- Patients with severe cognitive disorders which limit the understanding of the performing excesses

- Ambulant patients
- Patients who underwent surgery or suffered from a recent trauma of upper limbs (less than six months ago)
- Patients under corticosteroids during less than six months

For the controls

- Surgery or recent trauma of upper limbs(less than six months)
- High level athletes (national level)
- Chronic treatment impairing the muscular strength during the months before the inclusion.

5. Calendar and description of the evaluations

The patients and the control subjects will be evaluated using the Moviplate tool and other tests that help to verify the relevance of the former (Table 1). These additional tests are the following:

- Dynamometric measurements of muscular strength at the wrist (extension), hand (palmar grip) (Appendix 2) and fingers (key-pinch) level (Appendix 3)
- Taping-Test (Appendix 4)
- Functional Scales: Motor Function Measure (Appendix 5) (for patients only)
- Self-administered questionnaire (Functional status of a hand) (Appendix 6)

All the tests are for the upper limbs and will be conducted on both sides for each subject.

For the patients, the tests will approximately take sixty minutes; they will be conducted at the Institute of Myology, at the Raymond Poincaré Hospital, at the Armand Trousseau Hospital and the Necker Hospital. For the control subjects, the tests will approximately take thirty minutes and will be conducted at the Institute of Myology after obtaining the informed consent. The second evaluation will be done with using Moviplate and the other muscle strength tests (wrist, hand, fingers) and taping test on the same day, after at least an hour rest, or the latest in four weeks after the first visit (test duration estimated at 30 minutes).

After six months: patients (from the main diagnosis classes, DMD, SMA and sarcoglycannopathy) and controls will have a new visit to monitor the changes they have had since the inclusion in the study, and to repeat the same tests as at the inclusion (see table 1)

Table 1 : Evaluation Calendar

	**Patients**				**Control subjects**		
	**D0**	**<D30***	**D6months**	**D12 months**	**D0**	**<D30***	**D6months**
**MEDICAL SURGICAL HISTORY**	X	X	X	X	X	X	X
**Moviplate**	X	X	X	X	X	X	X
**MUSCULAR STRENGTH EVALUATION:**							
- wrist extension	X	X	X	X	X	X	X
- palmar grip	X	X	X	X	X	X	X
- key pinch force	X	X	X	X	X	X	X
TAPING	X	X	X	X	X	X	X
MFM	X		X	X			
Self-administered questionnaire (hand)	X		X	X	X		X
INTERCURRENT Evts		X	X	X		X	X
WEIGHT, HEIGHT, HR, BP	X	X	X	X	X	X	X

* The same day or the following day in thirty days after the inclusion.

For the patients, all the tests will be repeated in twelve months after the first visit.

- The Moviplate test (Appendix 1)

This test will be performed in the sitting position at the table where the Moviplate tool is placed (Appendix 1). This tool consists of a plateau with two small raised platforms which subjects have to touch alternately. Subjects perform the test in a sitting position with their forearm fixed or not fixed on the plateau placed before them on the table at the correct height. The purpose of the exercise is to make the maximum return motions in thirty seconds between the two platforms as a result of a coordinated extension movement of the wrist and fingers. The test is repeated two times at each evaluation (non-dominant side, dominant side then non-dominant side, dominant side).

- Dynamometrical measurements of muscular strength

Dynamometric measurements of the maximum strength of the following functions are performed: wrist extension (Myotools) (Appendix 2), palmar grip (Myotools) (Appendix 2) and key-pinch (pinch) (Appendix 3).

- Taping (Appendix 4)

The test includes typing on a strength sensor with the index finger as many times as possible in fifteen seconds. The energy used, the frequency, the number, the intensity and duration of contacts is studied over the time.

- Motor Function Measure (6, 7) (Appendix 5)

This scale gives a quantified measure of patient's motor skills. It includes thirty-two items, some are static and others are dynamic. The items are tested lying down, in sitting position, in standing position and they are divided into three dimensions and rated on a four-point scale (0 = test not performed, 3 = completely performed test):
-D1: standing position and transfers (thirteen items)
-D2: axial and proximal motor function (twelve items)
-D3: distal motor function (seven items)

- Self-administered questionnaire (performance status of a hand (9-10) (Appendix 6)

6. Data collection

The measuring results are collected in an experimental form given in Appendix 7.
All the tests are non-invasive and do not have any serious side effects. The risks associated with this study are related to forced movements (tiredness, muscle pain, muscle cramps, muscle or musculo-tendinous injuries related to stretching). The muscle evaluation will be stopped in case of occurrence of pain or cramp.

7. Expected profit

Profit is not expected out of the patient participating in this protocol.

8. Duration of the exclusion

The exclusion period is not expected after the inclusion of controls and patients. Neither extended follow-up**,** nor persistent effect expected.

9. Management of withdrawal from a trial

Subjects who cannot perform the tests described in the protocol or who do not want to follow the protocol for one reason or another will be replaced by another person of similar age.

10. Management of adverse effects

No serious adverse effects are expected in this study. As already pointed out, it is specified that the equipment used are only quantifiable tools that do not develop themselves any force, but remain passively resistant with a force equal to the force generated by the subject. There aren't any risks associated with the use of the tools (no invasive measurement, no electrical contacts). All the adverse effects will be marked, that is to say all the symptoms, clinical signs, reactions that develop or worsen during this study.

The information about all the adverse effects will be immediately written in the space provided for this purpose in the experimental forms and spread to the principal investigator as well as to the promoter. The evolution of each effect will be followed until recovery, stabilization, or until it is clearly established by mutual agreement that the effect is not related to the current study.

11. Investigator's commitments

The research will be conducted in compliance with the French regulations, including provisions related to biomedical research in the Public Health Code**,** Article L. 1121-1 and following, the Bioethics law, the information technology and liberties law (*CNIL*), , the Helsinki Declaration as well as the Recommendations for Good Practice and the present protocol.

The investigator agrees to conduct the research according to ethical norms and regulations. He is aware that all the documents as well as all the data related to the research may be the subject of audits and inspections in respect of professional confidentiality and may not be confronted with medical secrecy. The investigator recognizes that the study results are the property of the Institute of Myology, promoter of the research.

Before the implementation of the research, the coordinating investigator will put forward the project to the opinion of the Ethics Committee (*CPP*) Ile-de-France VI (Pitié-Salpêtrière - 47, Boulevard de l'Hôpital - 75651 PARIS Cedex 13) according to the Article L 209 of the Huriet law and will provide them with all the necessary information, the information notice and the consent form or other relevant document before being presented to the Committee. The trial will only begin when the Institute of

Myology has been informed of a favorable unreserved opinion issued by the CPP in regards to the protocol submitted. The promoter will inform the CPP about all the serious or unexpected adverse effects and appearance of new facts that could presumably affect people's safety.

The Institute of Myology, as the promoter, subscribed for the entire duration of the trail to an insurance contract №. .... according to French laws and regulations on biomedical research.

12. Information notice and informed consent

The written informed consent of people taking part in the research must be obtained by the investigator, enrolled in the medical association**,** declared as an investigator to the promoter before any act is performed on the basis of the research protocol, accordingly to regulations**.**

According to the law of December 20th, 1988 as amended, the information given orally and in writing to patients will include information about the purpose of research, methodology and its duration, the constraints and the foreseeable risks, the opinion of the CCP, as well as the right to refuse to participate in the research or to withdraw the consent at any time without any obligation.
Patients will give their free and informed consent in writing by signing and dating the consent form. This consent form will be prepared in two copies for the patient and for the physician-investigator so that each one keeps a copy.

The investigator must ensure that the person suitable for the research will have time to make a decision freely and will be able to read and understand the information notice and the consent form.
The information form of consent is a document that will be approved before the implementation of the research by the CPP, after examining the protocol.
No compensation is provided for patients under this protocol, except for refund of travel expenses.

13. Data management

13.1. Privacy policy

Personal data will remain confidential: the form with collected data will be referenced by a four-digit code (№ of center and № of included patient)

13.2 Data analysis

The reliability of the tests will be estimated by analyzing the correlation of intraclass correlation coefficient (ICC) and Bland and Altman analysis. The sensitivity to change will be assessed by the calculation of the differences between the evaluations at inclusion, at six and twelve months. To compare the sensitivity to change in Moviplate and in other tests, the Standardized Response Means (SRM) will be calculated by using group diagnosis taking into consideration the principal diagnosis (DMD, SMA, sarcoglycannopathy) and comparing them with the controls. The SRM values are interpreted using the following rule (11): low sensitivity (0.2 to 0.49), moderate (0.50 to 0.79), and high (≥ 0.80).

The correlations between the results of Moviplate and other tests will be analyzed for convergent validity.

The six-month evolution of the measures will be compared between patient and control groups by analyzing variation of repeated measures. The effects of age, sex, weight (and other morphological data) will be studied.

13.3 Archiving of the documents

Investigators will keep a file containing the list of the included subjects with the corresponding code, the copy of all the papers of the experimentation and all the documentation related to the study according to European regulations, for a period of fifteen years after the end of the study.

After data is stored digitally, the original documents will be kept by the investigator.

14. Amendments to the protocol

Neither the investigators nor the promoter will change the protocol without the consent of the second party.

Any substantial changes in the protocol (for example: the change in the course of the study, extension of the duration of the study, etc.) should be the subject of an amendment signed by the principal investigator and the promoter. It will be submitted by the CPP. The amendment signed and approved will be acknowledged and spread to all the collaborators of the research.

15. Duration of the study

The total study duration will take thirty six months. The duration of subjects' inclusion will

approximately take twenty four months (between September 2099 and September 2012) according to the reached agreements. As a reminder, the subjects are included in the protocol for about thirty –sixty minutes during the day.

16. Final report and publications

At the end of the study, a report will be prepared by the principal investigator in collaboration with various participants. It may result in a written, oral or poster presentations. According to the Public Health Code, each investigator or participant cannot publish or report the results of the study without the formal agreement with others and informing the promoter.

17. Bibliographie

1. Engel AG & Ozawa E. Dystrophinopathies, In Myology, third ed., Engel AG & Franzini-Armstrong C, Mc Graw-Hill, NY 2004. p 961-1026

2. Rudnick-Scöenberg S, de Visser M, Zeros K. Spinal Muscular Atrophies. In Myology, third ed., Engel AG & Franzini-Armstrong C, Mc Graw-Hill, NY 2004. p 1845-1864.

3. Mercuri, E., Mayhew, A., Muntoni, F., Messina, S., Straub, V., Van Ommen, G.J., Voit, T., Bertini, E., Bushby, K. (2008). Towards harmonisation of outcome measures for DMD and SMA within TREAT-NMD; report of three expert workshops: TREAT-NMD/ENMC workshop on outcome measures, 12th--13th May 2007, Naarden, The Netherlands; TREAT-NMD workshop on outcome measures in experimental trials for DMD, 30th June--1st July 2007, Naarden, The Netherlands; conjoint Institute of Myology TREAT-NMD meeting on physical activity monitoring in neuromuscular disorders, 11th July 2007, Paris, France. *Neuromuscul. Disord. 18*, 894-903.

4. ATS statement: guidelines for the six-minute walk test. Am J Respir Crit Care Med 2002; 166 (1): 111-117.

5. Main, M., Kairon, H., Mercuri, E. and Muntoni, F. (2003). The Hammersmith functional motor scale for children with spinal muscular atrophy: a scale to test ability and monitor progress in children with limited ambulation. *Eur. J. Paediatr. Neurol. 7*, 155-159.

6. Bérard, C., Payan, C., Hodgkinson, I., Fermanian, J. (2005). A motor function measure for neuromuscular diseases. Construction and validation study. *Neuromuscul. Disord.15*, 463-470.

7. Bérard, C., Payan, C., Fermanian, J., Girardot, F. (2006). [A motor function measurement scale for neuromuscular diseases - description and validation study]. *Rev. Neurol. (Paris) 162*, 485-493.

8. Mattar, F.L. and Sobreira, C. (2008). Hand weakness in Duchenne muscular dystrophy and its relation to physical disability. *Neuromuscul. Disord. 18*, 193-198.

9. Sezer N, Yavuzer G, Sivrioglu K,Basaran P, Koseoglu F. (2007). Clinimetric properties of the Duruoz Hand Index in patient with stroke. *Arch. Phys. Med. Rehabil.* 88 : 309-314.

10. Poireaudeau S, Lefèvre-Colau MM, Fermanian J, Mayoux-Benhamou MA, Revel M. (2000). Qualités métrologiques de l'indice d'incapacité fonctionnelle de Cochin adapté à la main rhumatoïde. *Ann. Réadaptation Med Phys.* 43 : 106-115.

11 . Bjorgvinsson T, Kerr P. (1995) Use of a common language effect size statistic. Am. J. Psychiatry 152:151.

18. Annexes

<u>Annexe 1 : Outil Moviplate</u>

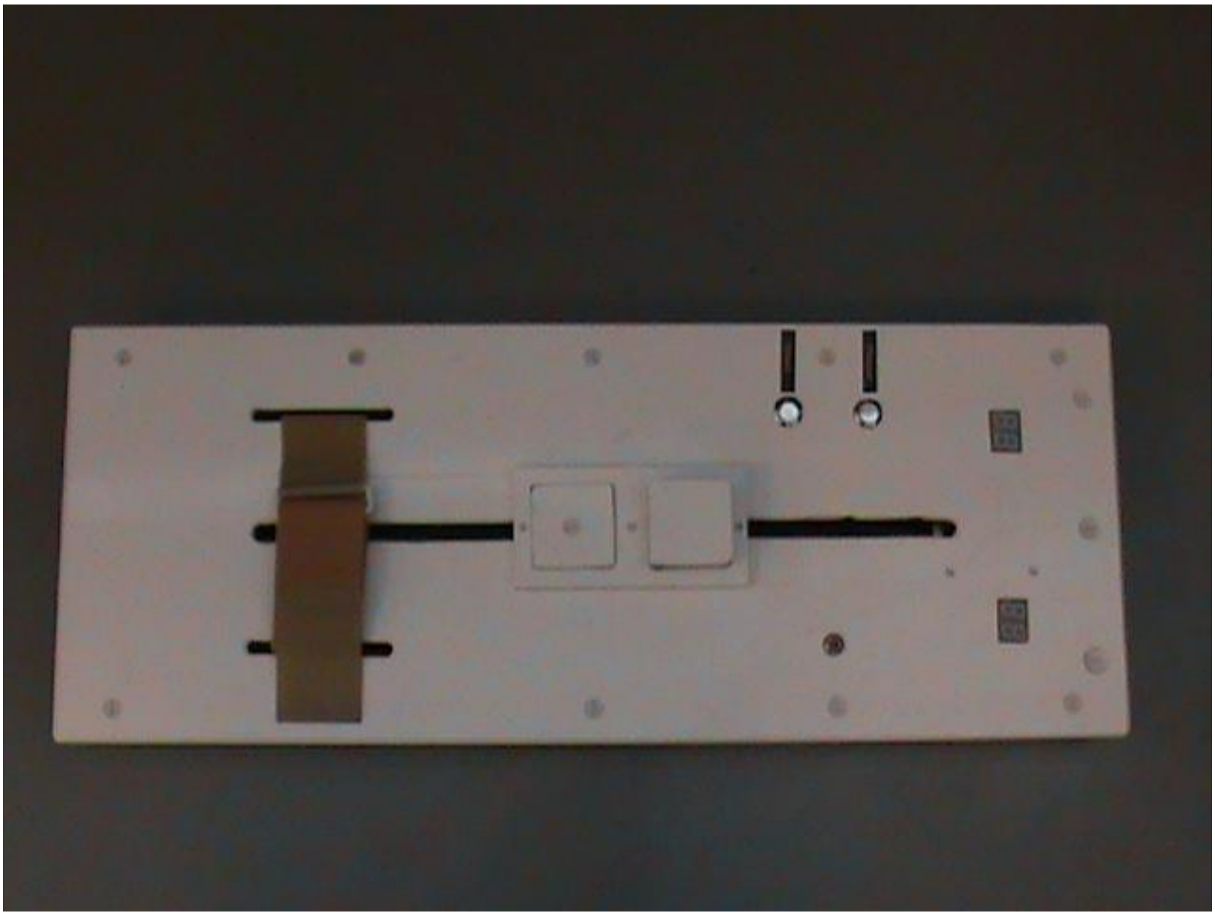

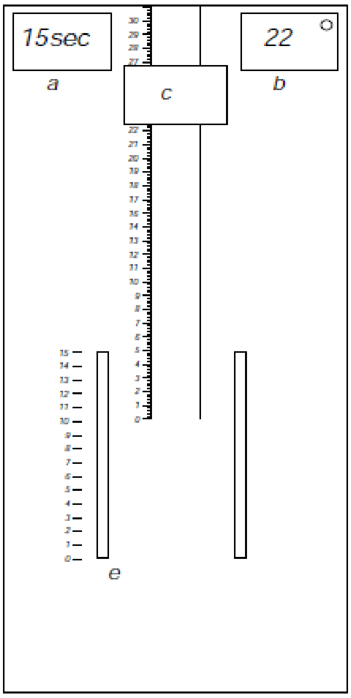

a : Chronomètre. En seconde, option compteur et décompteur, écran visualisable ou non.

b : Compteur. En valeur absolue, décompte visualisable. Il compte le nombre de contact avec c

c : Plateforme d’enregistrement, réglable en hauteur (1 ou 2 cm de hauteur), et ajustable

sur la longueur de la planche (cf schéma). Elle permet d’enregistrer sans analyse préalable la valeur discrète constituée par le nombre de contacts avec c pendant la durée du chronométrage.

d : Pieds

e : Fente pour ajustement de lanière de contention de l’avant bras.

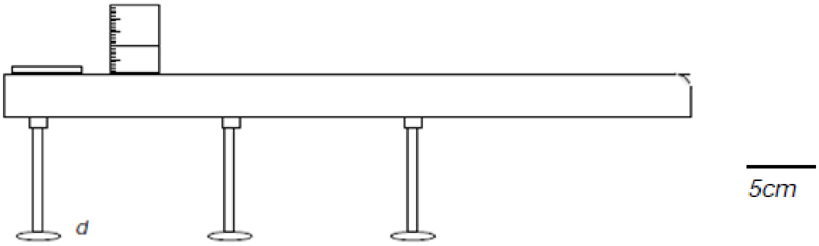

Annexe 2: outils Myotools

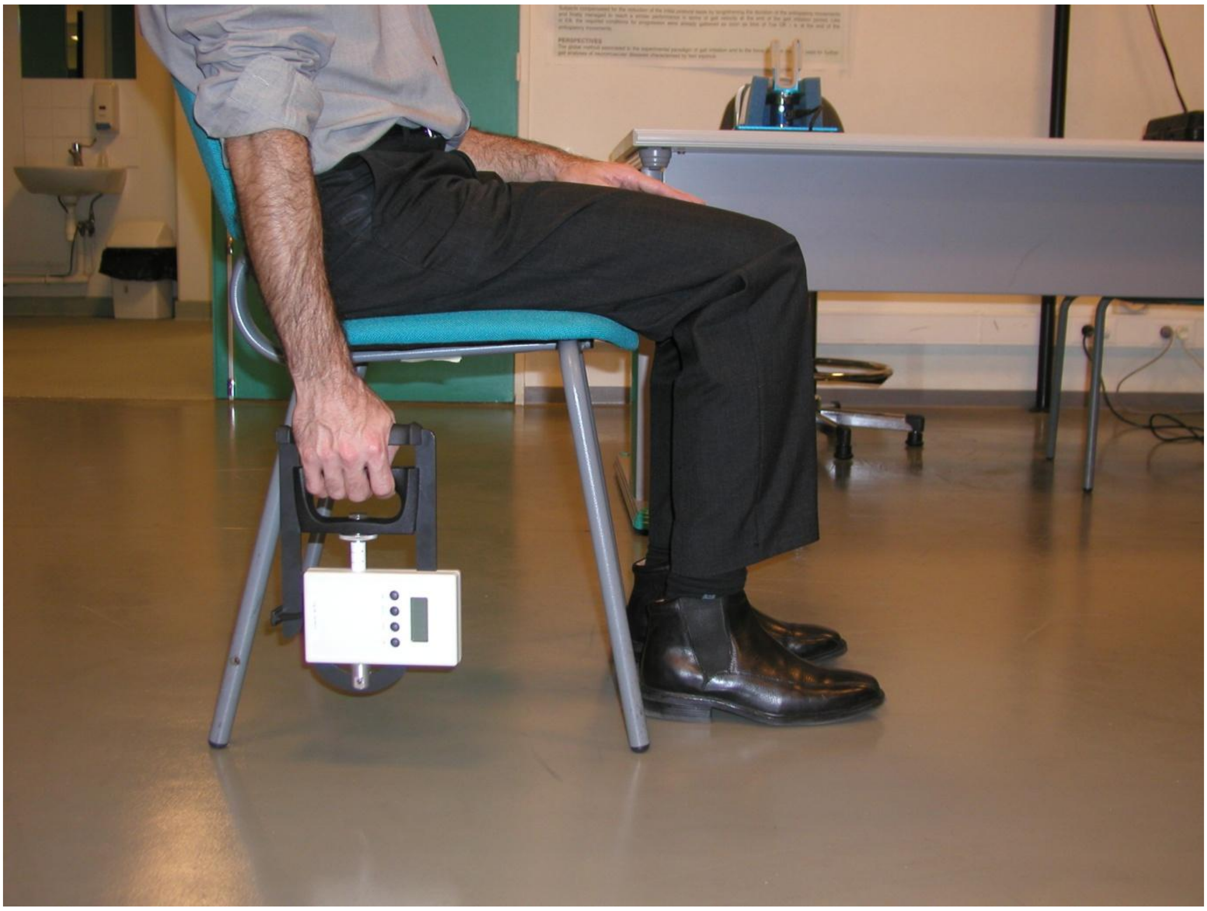

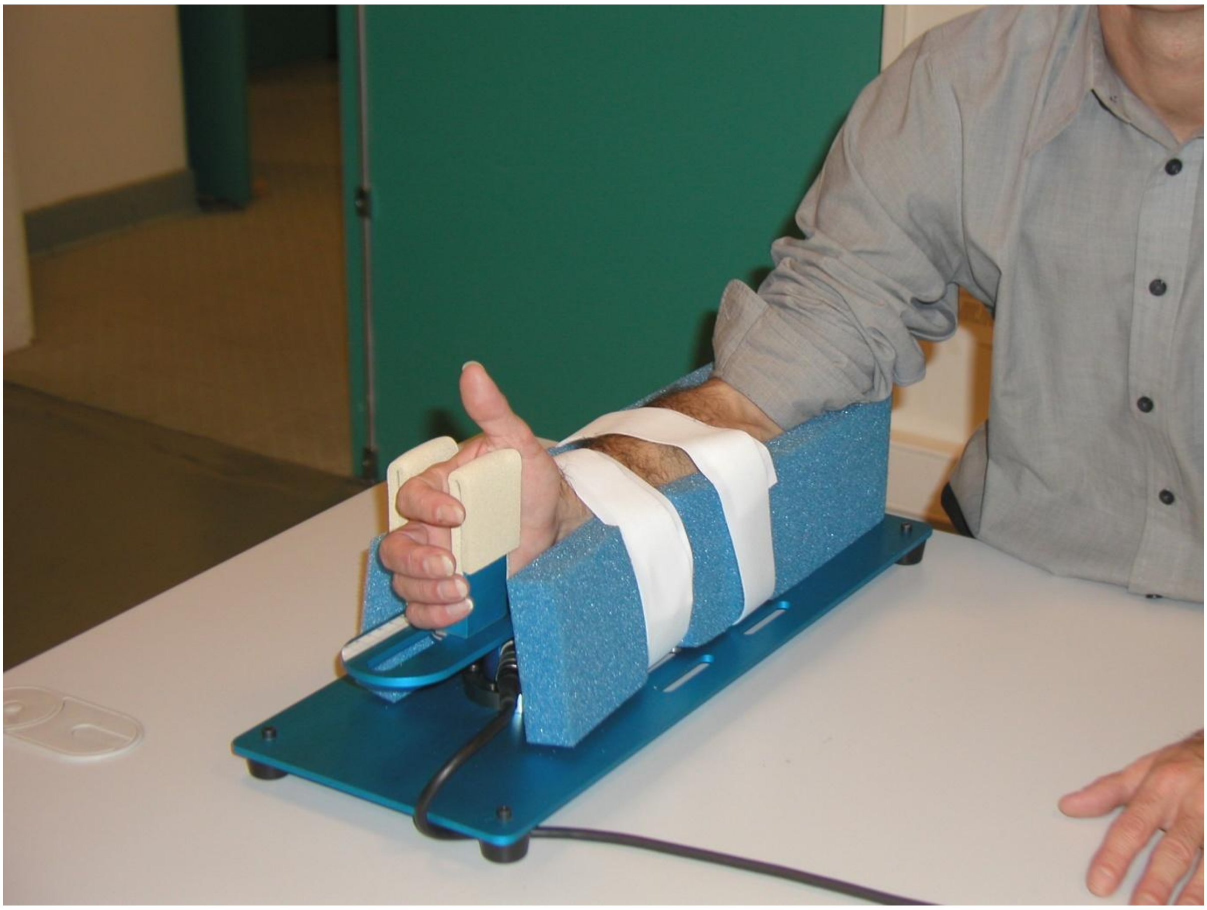

Annexe 3: Pinch

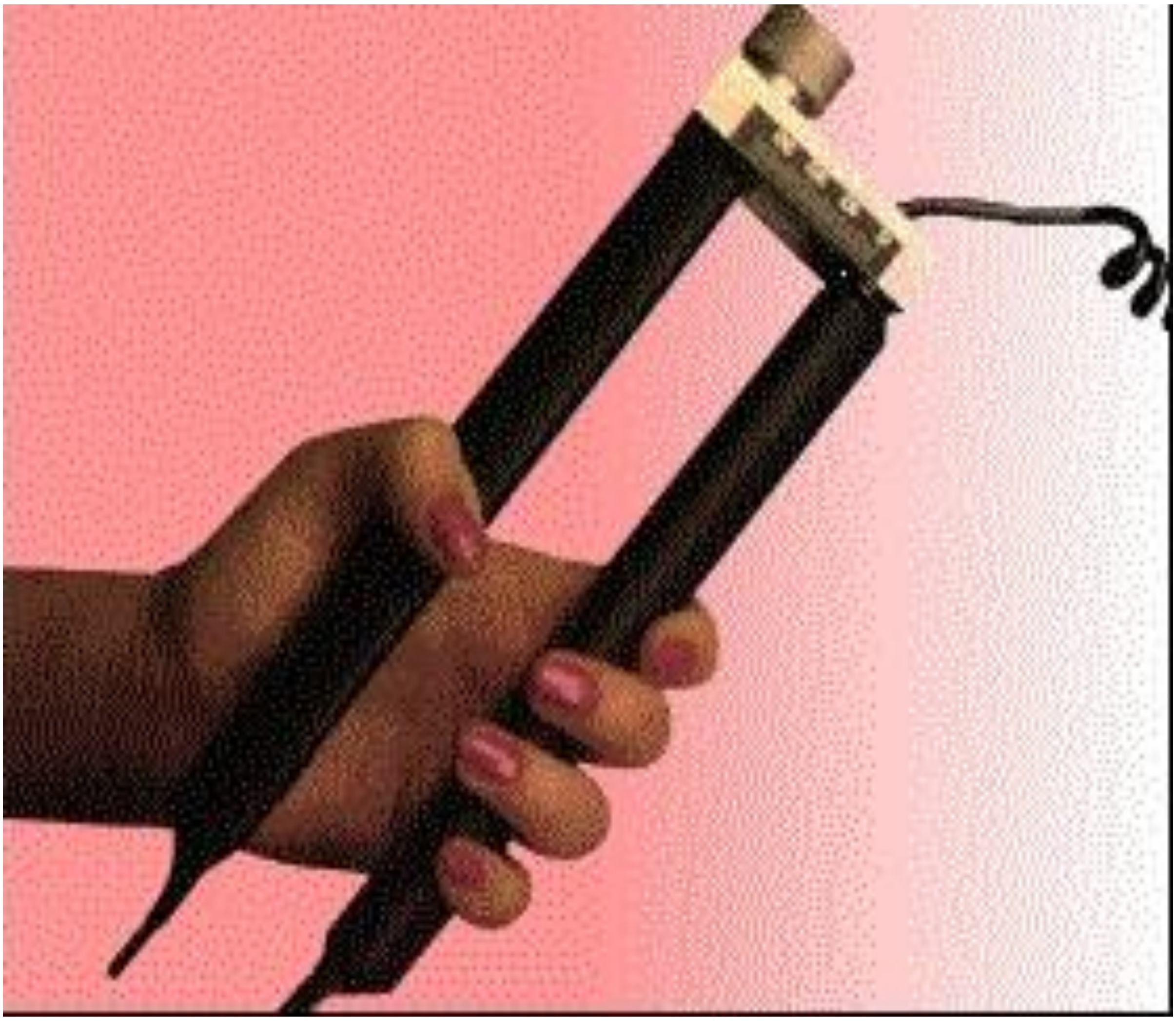

Annexe 4: Taping

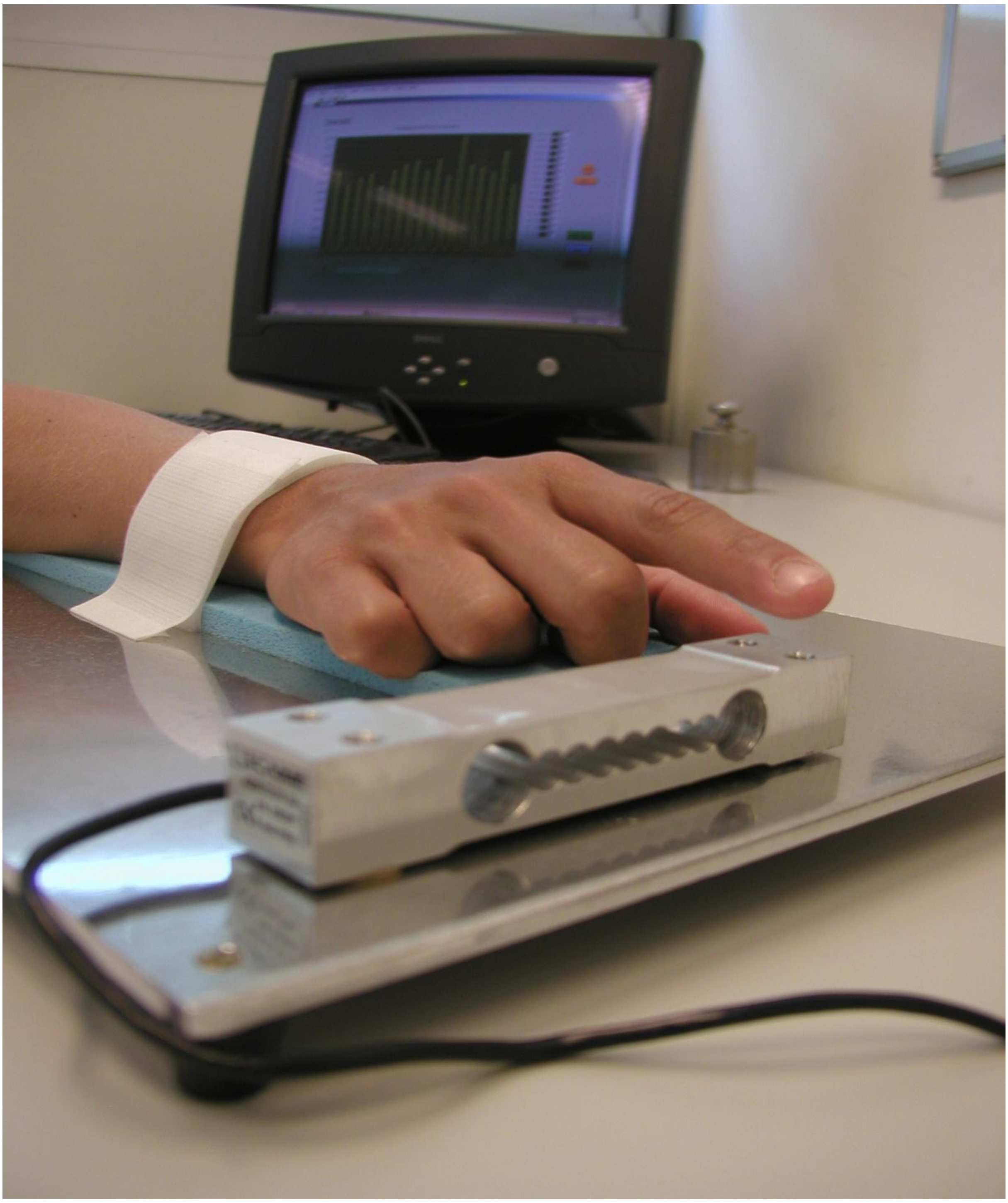

Annexe 5 : Echelle de Mesure de Fonction Motrice (MFM)

Cotation

**Items de l'échelle MFM**	**D1 =**	**D2 =**	**D3 =**
1. Couché sur le dos, tête dans l'axe : maintient la tête dans l'axe 5 secondes puis la tourne complètement d'un côté puis de l'autre<br>*commentaires* :		□ 0<br>□ 1<br>□ 2<br>□ 3	
2. Couché sur le dos : soulève la tête et la maintient soulevée 5 secondes.<br>*commentaires* :		□ 0<br>□ 1<br>□ 2<br>□ 3	
3. Couché sur le dos : fléchit la hanche et le genou de plus de 90° en soulevant le pied durant tout le mouvement.<br>côté choisi : droit 1 : □<br>gauche 2 : □<br>cotation controlatérale : □0 □1 □2 □3<br>*commentaires* :		□ 0<br>□ 1<br>□ 2<br>□ 3	
4. Couché sur le dos, jambe soutenue par l'examinateur : de la position pied en flexion plantaire, réalise une flexion dorsale du pied à au moins 90° par rapport à la jambe.<br>côté choisi : droit 1 : □<br>gauche 2 : □<br>cotation controlatérale : □0 □1 □2 □3<br>*commentaires* :			□ 0<br>□ 1<br>□ 2<br>□ 3
5. Couché sur le dos : soulève la main et la porte jusqu'à l'épaule opposée.<br>côté choisi : droit 1 : □<br>gauche 2 : □<br>cotation controlatérale : □0 □1 □2 □3<br>*commentaires* :		□ 0<br>□ 1<br>□ 2<br>□ 3	
6. Couché sur le dos, membres inférieurs demi fléchis, rotules au zénith, pieds sur le tapis légèrement écartés: maintient 5 secondes la position de départ puis soulève le bassin ; la colonne lombaire, le bassin et les cuisses sont alignés et les pieds légèrement écartés.<br>*commentaires* :	□ 0<br>□ 1<br>□ 2<br>□ 3		

Items de l'échelle MFM	D1 =	D2 =	D3 =
7. Couché sur le dos : se retourne sur le ventre et dégage les membres supérieurs de dessous le tronc.<br>côté choisi : droit 1 : ☐ gauche 2 : ☐<br>cotation controlatérale : ☐0 ☐1 ☐2 ☐3<br>*commentaires* :		☐ 0<br>☐ 1<br>☐ 2<br>☐ 3	
8. Couché sur le dos : sans appui des membres supérieurs, s'assied.<br>*commentaires* :	☐ 0<br>☐ 1<br>☐ 2<br>☐ 3		
9. Assis sur le tapis : sans appui des membres supérieurs, maintient 5 secondes la station assise puis maintient 5 secondes un contact entre les 2 mains.<br>*commentaires* :		☐ 0<br>☐ 1<br>☐ 2<br>☐ 3	
10. Assis sur le tapis, la balle de tennis devant le sujet : sans appui des membres supérieurs se penche en avant, touche la balle puis se redresse.<br>..<br>..<br>*commentaires* :		☐ 0<br>☐ 1<br>☐ 2<br>☐ 3	
11. Assis sur le tapis : sans appui des membres supérieurs, se met debout.<br>*commentaires* :	☐ 0<br>☐ 1<br>☐ 2<br>☐ 3		
12. Debout : sans appui des membres supérieurs, s'assied sur la chaise en gardant les pieds légèrement écartés.<br>*commentaires* :	☐ 0<br>☐ 1<br>☐ 2<br>☐ 3		
13. Assis sur la chaise : sans appui des membres supérieurs et sans appui contre le dossier de la chaise, maintient 5 secondes la position assise, tête et tronc dans l'axe.<br>*commentaires* :		☐ 0<br>☐ 1<br>☐ 2<br>☐ 3	
14. Assis sur la chaise ou dans son fauteuil, tête positionnée en flexion : de la position tête fléchie complètement, relève la tête puis la maintient relevée 5 secondes, le mouvement et le maintien se faisant tête dans l'axe.<br>*commentaires* :		☐ 0<br>☐ 1<br>☐ 2<br>☐ 3	
15. Assis sur la chaise ou dans son fauteuil, avant-bras posés sur la table, coudes en dehors de la table : porte en même temps les 2 mains sur le sommet du crâne, la tête et le tronc restant dans l'axe.<br>*commentaires* :		☐ 0<br>☐ 1<br>☐ 2<br>☐ 3	

Items de l’échelle MFM	D1 =	D2 =	D3 =
16. Assis sur la chaise ou dans son fauteuil, le crayon sur la table : sans bouger le tronc, atteint le crayon avec la main, avant-bras et main soulevés de la table, coude en extension complète en fin de mouvement.<br>côté choisi : droit 1 : □ gauche 2 : □<br>cotation controlatérale : □0 □1 □2 □3<br>*commentaires* :		□ 0<br>□ 1<br>□ 2<br>□ 3	
17. Assis sur la chaise ou dans son fauteuil, 10 pièces de monnaie sur la table : prend successivement et stocke 10 pièces dans une main au bout de 20 secondes.<br>côté choisi : droit 1 : □ gauche 2 : □<br>cotation controlatérale : □0 □1 □2 □3<br>*commentaires* :			□ 0<br>□ 1<br>□ 2<br>□ 3
18. Assis sur la chaise ou dans son fauteuil, un doigt posé au centre d'un CD fixe : fait le tour du CD avec le même doigt, sans appui de la main.<br>côté choisi : droit 1 : □ gauche 2 : □<br>cotation controlatérale : □0 □1 □2 □3<br>*commentaires* :			□ 0<br>□ 1<br>□ 2<br>□ 3
19. Assis sur la chaise ou dans son fauteuil, le crayon sur la table : prend le crayon puis dessine une série continue de boucles sur toute la longueur du cadre touchant les bords supérieur et inférieur du cadre.<br>côté choisi : droit 1 : □ gauche 2 : □<br>cotation controlatérale : □0 □1 □2 □3<br>*commentaires* : ....<br>**Essai n°1** essai controlatéral n°1<br>**Essai n°2** essai controlatéral n°2			□ 0<br>□ 1<br>□ 2<br>□ 3
20. Assis sur la chaise ou dans son fauteuil, la feuille de papier dans les mains : déchire la feuille pliée en 4 en commençant par le pli.<br>*commentaires* :			□ 0<br>□ 1<br>□ 2<br>□ 3

Items de l'échelle MFM	D1 =	D2 =	D3 =
21. Assis sur la chaise ou dans son fauteuil, la balle de tennis sur la table : soulève la balle puis retourne la main complètement en tenant la balle.<br>côté choisi : droit 1 : □ gauche 2 : □<br>cotation controlatérale : □0 □1 □2 □3<br>*commentaires* :			□ 0<br>□ 1<br>□ 2<br>□ 3
22. Assis sur la chaise ou dans son fauteuil, un doigt posé au centre du carré: soulève le doigt puis le pose successivement dans les 8 carrés de la figure sans toucher le quadrillage.<br>côté choisi : droit 1 : □ gauche 2 : □<br>cotation controlatérale : □0 □1 □2 □3<br>*commentaires* :			□ 0<br>□ 1<br>□ 2<br>□ 3
23. Assis sur la chaise ou dans son fauteuil, membres supérieurs le long du corps : pose en même temps les 2 avant-bras et/ou les mains sur la table sans bouger le tronc.<br>*commentaires* :		□ 0<br>□ 1<br>□ 2<br>□ 3	
24. Assis sur la chaise : sans appui des membres supérieurs, se met debout les pieds légèrement écartés.<br>*commentaires* :	□ 0<br>□ 1<br>□ 2<br>□ 3		
25. Debout avec appui des membres supérieurs sur un matériel : sans appui des membres supérieurs, maintient 5 secondes la position pieds légèrement écartés, tête, tronc et membres inférieurs dans l'axe.<br>*commentaires* :	□ 0<br>□ 1<br>□ 2<br>□ 3		
26. Debout avec appui des membres supérieurs sur un matériel : sans appui des membres supérieurs, lève un pied 10 secondes.<br>côté choisi : droit 1 : □ gauche 2 : □<br>cotation controlatérale : □0 □1 □2 □3<br>*commentaires* :	□ 0<br>□ 1<br>□ 2<br>□ 3		
27. Debout : sans appui, touche le sol avec une main puis se relève.<br>*commentaires* :	□ 0<br>□ 1<br>□ 2<br>□ 3		
28. Debout sans appui : fait 10 pas en avant sur les 2 talons.<br>*commentaires* :	□ 0<br>□ 1<br>□ 2<br>□ 3		
29. Debout sans appui : fait 10 pas en avant sur une ligne droite.<br>*commentaires* :	□ 0<br>□ 1<br>□ 2<br>□ 3		

Items de l'échelle MFM	D1 =	D2 =	D3 =
30. Debout sans appui : court 10 mètres.<br>*commentaires* :	☐ 0<br>☐ 1<br>☐ 2<br>☐ 3		
31. Debout sur un pied sans appui : saute sur un pied 10 fois de suite sur place.<br>côté choisi : droit 1 : ☐ gauche 2 : ☐<br>cotation controlatérale : ☐0 ☐1 ☐2 ☐3<br>*commentaires* :	☐ 0<br>☐ 1<br>☐ 2<br>☐ 3		
32. Debout sans appui : sans appui des membres supérieurs, atteint la position accroupie puis se relève 2 fois de suite.<br>*commentaires* :	☐ 0<br>☐ 1<br>☐ 2<br>☐ 3		
**TOTAL**	**D1=**	**D2=**	**D3=**

Durée de la passation : I___I___I___I Minutes

Coopération du patient : nulle I___I 0, moyenne I___I 1, optimale I___I 2

Commentaires sur le déroulement du test et sur les résultats obtenus: (*texte libre, si rien de particulier, noter RAS*)
………
………
…………………………………………...

Annexe 6 : Auto-questionnaire (Indice fonctionnel de la main)

Réponses aux questions : 0 = oui sans difficulté ; 1 = possible avec très peu de difficultés ; 2 = possible avec quelques difficultés ; 3 = possible avec beaucoup de difficultés ; 4 = presque impossible ; 5 = impossible.
Veuillez répondre aux questions ci-dessous, sans appareillage adapté :

C1 – A la cuisine
Pouvez-vous tenir un bol ?
Pouvez-vous saisir une bouteille pleine et la lever ?
Pouvez-vous tenir un plat plein ?
Pouvez-vous verser le liquide de la bouteille dans un verre ?
Pouvez-vous dévisser le couvercle d'un pot déjà ouvert une fois ?
Pouvez-vous couper la viande avec un couteau ?
Pouvez-vous piquer efficacement avec une fourchette ?
Pouvez-vous peler des fruits ?

C2 – Habillage
Pouvez-vous boutonner votre chemise
Pouvez vous ouvrir puis fermer les fermetures éclairs ?

C3 – Toilette
Pouvez-vous presser un tube de dentifrice avec un crayon ou un stylo ordinaire ?
Pouvez-vous tenir votre brosse à dent efficacement ?

C4 – Au bureau
Pouvez-vous écrire une phrase courte avec un crayon ou un stylo ordinaire ?
Pouvez-vous écrire une lettre avec un crayon ou un stylo ordinaire ?

C5 – Divers
Pouvez-vous tourner une poignet de porte ronde ?
Pouvez-vous utiliser des ciseaux pour couper un morceau de papier ?
Pouvez-vous saisir les pièces de monnaie sur une table ?
Pouvez-vous tourner une clé dans la serrure ?

Annexe 7 : Fiche expérimentale

Code sujet : |__|__|__| |__|__|__| |__|__|

Date de la visite : |__|__|-|__||__|-|__|__|__|__| Heure de la visite : |__|__|:|__|__|

Visite n° |__|

Date de naissance : |__|__|-|__||__|-|__|__|__|__|

Poids : |__|__|__|,|__| kg Taille : |__|__|__|,|__| cm %ge Masse grasse : |__|__|,|__|

Côté Dominant : Gauche ☐ Droit ☐ Ambidextre ☐

Circonférence avant-bras |__|__|,|__| cm Circonférence main |__|__|,|__| cm
Taille main |__|__|,|__| cm

Observations (antécédents traumatiques, douleurs, pathologies de l'appareil locomoteur...)
…………………………………………………………………………………………………
…………………………………………………………………………………………………
……………………………………………………………………..

Moviplate Evaluateur (………...…….)

Position de la cible |__|__|,|__| cm
Hauteur de la cible 1cm☐ 2cm ☐

Avant-bras fixé :
Position de la sangle de fixation |__|__|,|__| cm

	1. heure |__|__|:|__|__|	2. heure |__|__|:|__|__|
Nb touchers de cible (30s)	G |__|__| D |__|__|	G |__|__| D |__|__|

Commentaires :
…………………………………………………………………………………………………
………………………………………………………………………………

Avant-bras libre :
Position de l'appui levier de l'avant-bras |__|__|,|__| cm de la pointe inférieure de la styloïde radiale

	1. heure |__|__|:|__|__|	2. heure |__|__|:|__|__|
Nb touchers de cible (30s)	G |__|__| D |__|__|	G |__|__| D |__|__|

Commentaires :
…………………………………………………………………………………………………
………………………………………………………………………………

Préhension palmaire (poignée IDM) **Evaluateur (…....................…...)**

Largeur poignée: |__|,|__|

		1	2	3	4	5
G	MVC (daN)	|__|__|,|__|__|	|__|__|,|__|__|	|__|__|,|__|__|	|__|__|,|__|__|	|__|__|,|__|__|
D	MVC (daN)	|__|__|,|__|__|	|__|__|,|__|__|	|__|__|,|__|__|	|__|__|,|__|__|	|__|__|,|__|__|

Commentaires :
………………………………………………………………………………………………………
………………………………………………………………………………

Extension de poignet (outil Myotools) **Evaluateur (……………….…...)**

Distance appui palmaire : |__|__|,|__| cm
Hauteur table : |__|__|,|__| cm

		1	2	3	4	5
G	MVC (N.m)	|__|__|,|__|__|	|__|__|,|__|__|	|__|__|,|__|__|	|__|__|,|__|__|	|__|__|,|__|__|
D	MVC (N.m)	|__|__|,|__|__|	|__|__|,|__|__|	|__|__|,|__|__|	|__|__|,|__|__|	|__|__|,|__|__|

Commentaires :
………………………………………………………………………………………………………
………………………………………………………………………………

Pince pouce-index (pinch) **Evaluateur (……………....…...)**

		1	2	3	4	5
G	MVC (kg)	|__|__|,|__|__|	|__|__|,|__|__|	|__|__|,|__|__|	|__|__|,|__|__|	|__|__|,|__|__|
D	MVC (kg)	|__|__|,|__|__|	|__|__|,|__|__|	|__|__|,|__|__|	|__|__|,|__|__|	|__|__|,|__|__|

Commentaires :
………………………………………………………………………………………………………
………………………………………………………………………………

Taping **Evaluateur (……………....…...)**
Réglages
Nombre de taps (15 secondes) |__|__|__|

Commentaires :
………………………………………………………………………………………………………
………………………………………………………………………………
